# Conscientious objection in euthanasia and assisted suicide: A systematic review

**DOI:** 10.1371/journal.pone.0326142

**Published:** 2025-06-23

**Authors:** Carlos Gomez-Virseda, Chris Gastmans

**Affiliations:** Centre for Biomedical Ethics and Law, Department of Public Health & Primary Care, KU Leuven, Leuven, Belgium; University of Granada: Universidad de Granada, SPAIN

## Abstract

**Introduction:**

As euthanasia and assisted suicide (EAS) become legal in more countries, conscientious objection (CO) of healthcare professionals is gaining increasing attention. While some argue that CO safeguards professionals’ moral integrity, others view it as a barrier to patients’ access to desired healthcare. This review provides a comprehensive synthesis of the ethical literature regarding CO to EAS and answers three key questions: What is the meaning of CO and how is it used in EAS? What ethical positions support or challenge it? What underlying presuppositions shape the debate?.

**Methods:**

We used the PRISMA guidelines, RESERVE standards, and TARCiS statement to conduct a systematic review of argument-based publications retrieved from 13 major databases covering biomedical, philosophical, and theological literature. No date or language restrictions were applied. Titles and abstracts were independently screened by the two authors, and complete articles were selected based on predefined inclusion and exclusion criteria.

**Results:**

We identified 58 pertinent articles that were included in our review. Of these, 51 were published in the last decade, from 2015 through 2024. Our findings highlight three key dimensions. First, while there is general agreement on the definition of CO, its interpretation and application in EAS remain highly contested. Second, the ethical debate revolves around three main positions: conscience absolutism, the compromise approach, and the incompatibility thesis. Each of these is supported by distinct ethical arguments. Third, the debate is shaped by several underlying presuppositions, including divergent views on conscience, morality, religion, medicine, and end-of-life care.

**Conclusions:**

Our results highlight the risk of polarization in the debate on CO in EAS. It emphasizes the importance of dialogue between theoretical and context-sensitive perspectives to support more effective implementation of CO. Clearer guidelines are needed to balance respect for conscience, patient rights, and professional responsibilities in this complex issue.

## Introduction

As the legalization of euthanasia and assisted suicide (EAS) continues to expand globally [1], conscientious objection (CO) in healthcare has become an increasingly significant ethical issue [2–4]. Healthcare professionals face ethical dilemmas regarding their participation in EAS, as some perceive these practices to conflict with their deeply held moral convictions and often seek exemptions from legally authorized EAS procedures [5].

Public discourse on CO risks becoming increasingly polarized. Advocates argue for the accommodation of CO, emphasizing the importance of respecting healthcare professionals’ moral integrity [4,5]. In contrast, critics stress that professional obligations should take precedence over personal beliefs, warning that widespread accommodation of healthcare professionals’ CO could undermine equitable and efficient healthcare delivery [2,3]. They caution that CO can be invoked inconsistently or self-interestedly, and that it may place an outsized weight on the views of a religious minority, potentially privileging perspectives that do not align with core professional commitments [2]. In this context, a heated debate has emerged around the limits of CO, as some professionals appear to invoke it for reasons unrelated to deeply held moral convictions [6]. This highlights the need to clarify the concept of CO and distinguish it from other forms of non-participation in EAS.

Amid this polarized debate, academic interest in CO in end-of-life care has grown exponentially in recent years. While several reviews have synthesized the existing literature, many are outdated or lack methodological rigor [6–8]. Non-systematic searches of the extant literature often yield incomplete findings, leading to reviews that lack comprehensiveness or transparency. Moreover, an excessively broad scope can blur important distinctions, such as those between normative and empirical studies or distinctions between institutional and individual objections.

To address these gaps, we conducted a systematic review of theoretical literature to clarify (a) the meaning and uses of individual CO in the context of EAS; (b) ethical positions and arguments; and (c) underlying presuppositions that shape the debate.

## Methods

### Design

Systematic reviews have increasingly been proposed as a necessary approach in current bioethics. [9] Among them, systematic reviews of argument-based literature aim to provide up-to-date and comprehensive overviews of ethical concepts and arguments related to a certain topic. [10,11]. Following recent recommendations, our systematic review adhered to standardized methods to ensure transparency and reproducibility [12]. The review protocol was preregistered with PROSPERO (ID: CRD42024592004) and is available as Supporting information (S1 File). Its design and reporting adhered to the Preferred Reporting Items for Systematic Reviews and Meta-Analyses (PRISMA) statement [13], the REporting of SystEmatic ReViews in Ethics (RESERVE) standards [14], and the Terminology, Application, and Reporting of Citation Searching (TARCiS) recommendations [15]. Checklists for each protocol are provided as Supporting information, ensuring rigorous compliance with established best practices (S2 File-4).

### Research questions

The following questions were formulated in line with our research objectives:

1. What are the meaning and uses of CO among healthcare professionals in the context of EAS?
2. What are the main ethical positions and related arguments of CO in EAS?
3. What are the underlying presuppositions that shape the debate on CO in EAS?

### Literature search

To guide our literature search, we developed two sets of concept-related terms (Table 1): one for CO-related terminology and another for EAS practices, each encompassing legal and technical wording variations. Regarding the former, we included not only CO but also closely related phenomena that are sometimes conflated with it; however, our primary analytical focus remains on CO rather than other forms of non-participation. Regarding the latter, we treated CO in both euthanasia and physician-assisted suicide as a single category (EAS). Although these practices may prompt distinct ethical considerations due to differing levels of professional involvement, this choice reflects the legal heterogeneity across countries [1] and the fact that many argument-based contributions do not clearly distinguish between them.

**Table 1:** Groups of organizing concepts and associated database search terms^a^.

A. Conscientious objection	B. Euthanasia and assisted suicide
Conscientious; conscience; religious object*; moral object*; ethical object*; religious conflict*; moral conflict*; ethical conflict*; refusal*; exemption*; dilemma*; patient abandonment; contestant*; dissenter*; dissident*; objector*; protester*; non participation; non compliance	euthan*; mercy killing*; assisted suicide*; assisted dying; assistance in dying; assisted death*; death with dignity; dying with dignity; right to die; aid in dying; MAID; hastening death*; hastened death*; end of life decision*; end of life choice*; end of life right*; end of life option act*; life-ending intervention*; compassionate death*; life ending act*; life ending decision*; active life termination*; termination of life on request*

^a^Terms specific to each database Medical Subject Heading for Medline and “Deprescription” as a Embase Subject Heading (supplemented with relevant keywords identified in the literature).

Terms were drawn from controlled vocabulary specific to each database (e.g., Medical Subject Heading [MeSH] and Embase Subject Heading [Emtree] terms) and supplemented with relevant keywords identified in the literature. Both sets were subsequently translated into database-specific search terms and formatted for each query. The full search strategy was reviewed by an independent librarian from KU Leuven and is provided as Supporting information S5 File.

Thirteen electronic databases were queried, covering the fields of healthcare sciences, philosophy, and theology: *PubMed, Embase, Web of Science, CINAHL, SciELO, Scopus, ProQuest Central, Philosopher’s Index, JSTOR, Phil Papers, ATLA, Index Religiosus,* and *Index Theologicus*. Boolean searches were conducted in English across all databases.

The search was completed on September 19, 2024, with no date or language restrictions. Citations were imported into the citation management application EndNote 21.0 [16]. Duplicates were identified and removed using the software’s duplicate detection tool. Eligible articles were selected for analysis based on predefined inclusion and exclusion criteria (Table 2).

**Table 2:** Inclusion and exclusion criteria.

	Inclusion criteria	Exclusion criteria
Type of publication	Published articles in peer-reviewed academic journals	Non-peer-reviewed articles; non-academic sources (e.g., blogs or magazines); conference abstracts; preprints; book chapters or dissertations
	The publication is considered argument-based literature (i.e., a fully elaborated text that uses ethical concepts from contemporary or traditional philosophical theories) to construct arguments and address conceptual questions^a^	Empirical studies; opinion pieces (e.g., editorials); literature reviews; technical reports; guidelines; protocols, ethics policies, or ethics codes
Topic	Focus on individual conscientious objections of any clinical healthcare professional^b^	Focus on non-clinical health professionals^b^ or institutional conscientious objections^c^
	Substantially applied to euthanasia or assisted suicide (EAS) practices	Insufficient or unclear application to EAS practices

aDefinition based on McCullough [10] and Strech [11].

bIn line with the International Standard Classification of Occupations: ISCO-08, the World Health Organization differentiates health professionals (e.g., physicians, nurses, midwives, pharmacists and dentists) from health associate professionals, personal care workers in health services, health management and support personnel. We refer to the former as healthcare professionals and the latter as non-clinical health professionals.

cArticles focusing exclusively on institutional conscientious objections were excluded. For eligible articles addressing both institutional and individual objections, only the sections relevant to individual objections were included in our review.

**Fig 1:**
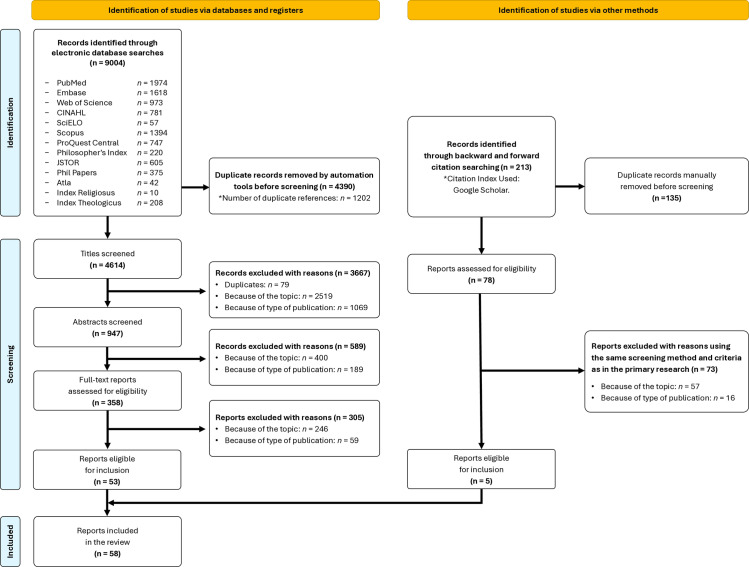
Flow chart illustrating the screening and selection of articles [13].

To ensure consistency, both authors independently screened titles and abstracts, achieving agreement rates of 92% for titles (4289/4614) and 89% for abstracts (843/947). The first author then assessed the full texts for inclusion eligibility. Throughout the screening process, each marginally acceptable article was discussed with the other author until a consensus was reached. To supplement the database searches, we performed backward and forward citation searching after full-text screening to identify additional publications not captured in the initial search. Following TARCiS recommendations, we iteratively repeated this process on newly identified eligible references until no further eligible studies were found (two iterations, with the final iteration performed on November 24, 2024) [15]. A PRISMA flow diagram detailing the screening and selection process is provided in Fig 1.

Given the absence of an established standard for quality appraisal of argument-based literature, we adopted Mertz’s strategy of “appraisal using procedural quality assurance criteria” [17]. Hence, we relied on the peer review process and the academic publisher’s reputation to ensure the adequacy of the included publications.

### Data extraction and synthesis

**Fig 2:**
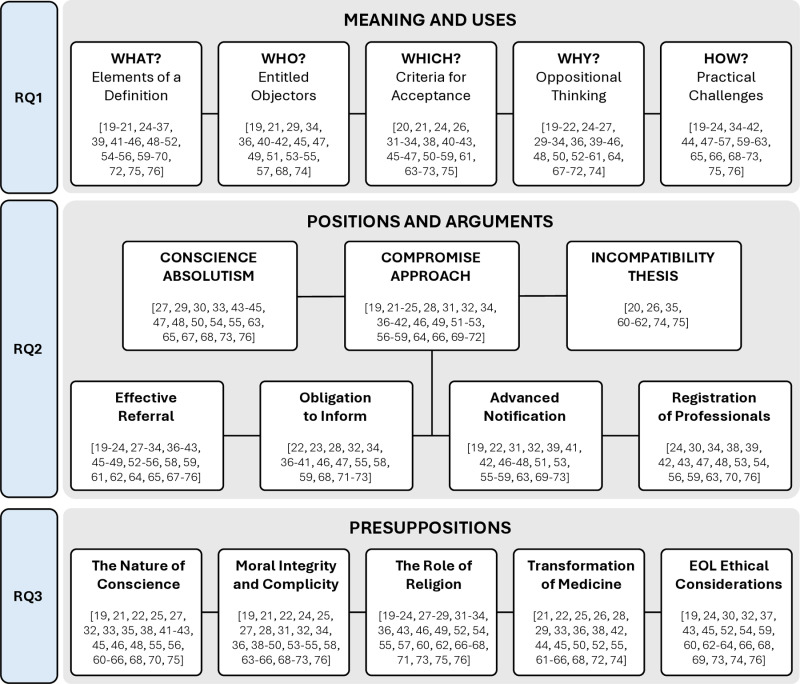
Three-level global scheme emerging from the analysis of the 58 included articles.

We adapted the five-step Qualitative Analysis Guide of Leuven (QUAGOL) [18] to a systematic review of argument-based literature approach and then applied it for data extraction and synthesis. First, we read the articles and highlighted relevant sections. Second, we drafted a narrative summary to identify key concepts and arguments. Third, we created a conceptual scheme for each publication, linking ethical concepts and arguments to our research questions (an example of a conceptual scheme is provided as Supporting information S6 File). These schemes were independently reviewed and refined through discussion until consensus was achieved. Fourth, we collectively analyzed the individual schemes and developed a three-layered global scheme, with each level corresponding to one of our research questions (Fig 2). Finally, we synthesized these findings into a report presented in the Results section. To enhance readability, we provide key references from articles that address topics in depth, rather than listing every article that mentions them. More detailed lists of references are available in the tables and figures.

## Results

Fifty-eight articles met our inclusion criteria and were analyzed to answer our research questions [19–76]. Their key characteristics are summarized in Table 3.

**Table 3:** Description of characteristics of included publications.

Analyzed features	Number of publications
JOURNAL	
**➢ **Language****	
English	51
Spanish	4
Dutch, French, German	1 *(each language)*
**➢ Year of publication**	
1990-1994	1
1995-1999	2
2000-2004	3
2005-2009	0
2010-2014	2
2015-2019	24
2020-2024	26
**➢ Academic discipline**	
Bioethics	29
Law	12
Medicine	5
Theology	4
Philosophy	3
Nursing	3
Pharmacy	2
FIRST AUTHOR	
**➢ Country of professional affiliation**	
Canada	17
USA	12
UK	8
Australia, Spain	5 *(each country)*
Belgium, Italy	2 *(each country)*
Colombia, Germany, Ireland, Mexico, Norway, Poland, South Africa	1 *(each country)*
**➢ Professional background**	
Medicine	21
Philosophy	21
Law	11
Nursing	4
Theology	1
CONTENT	
**➢ Ethical approach**	
Deontology	12
Rights-based ethics	10
Teleological and virtue ethics	8
Utilitarian and consequentialist ethics	7
Relational and care ethics	6
Public liberalism	4
Personalist ethics	3
Principled-based ethics	3
Professional ethics	3
Contextual ethics	2
**➢ CO ethical position**	
Compromise approach	32
Conscience absolutism	18
Incompatibility thesis	8
**➢ End-of-life focus**	
Both Euthanasia and Assisted suicide	37
Assisted suicide	16
Euthanasia	5
**➢ Main objector**	
Physicians	29
Healthcare professionals (in general)	23
Nurses	3
Pharmacist	3

Most of the included publications were published in the last decade, between 2015 and 2024 (n = 50), reflecting the increasing focus and interest in the topic. While the majority were published in English (n = 51), the authors were affiliated with institutions across diverse regions worldwide. The first authors predominantly had medical, philosophical, or legal backgrounds. Academic journals dealing with bioethics were the most common publication outlet (n = 29), underscoring the multidisciplinary nature of the field of bioethics. Finally, the included articles covered a broad range of ethical perspectives, addressing all relevant positions on CO, diverse EAS practices, and potential objectors to CO.

We now present our findings, organized into three levels, centered around our research questions. This organization is illustrated in our global scheme (cf. Fig 2). The first level explores the *meaning and uses* of CO in the context of EAS. The second level outlines the main *ethical positions* on CO in EAS and examines the arguments supporting these perspectives. The third level delves into *the underlying presuppositions* that shape the debate on CO in EAS.

### Meaning and uses of CO

Our first research question aimed to define CO and analyze its use in the ethical literature dealing with EAS. This question can be divided into the following five sub-questions: (a) *What* is CO in the context of EAS? (b) *Who* can object to EAS practices? (c) *Which* objections are acceptable? (d) *Why* is CO ethically contentious? (e) *How* is CO applied in EAS?

### *What* is CO in the context of EAS?

The term *conscientious objection* (CO) was the most frequently used across the analyzed literature; however, related terms such as non-participation [42,56], refusal to treat [19,31,32,61,65], patient abandonment [39,45], and professional dissent [55,67] were also identified. These terms sometimes appeared as interchangeable with CO, while in other cases they were conceptually distinguished. Clarifying the conceptual boundaries between CO and these related phenomena is important, as each reflects different motivations, ethical implications, and legal consequences [51,56,63,64]. In the reviewed articles, CO is typically grounded in deeply held moral or religious convictions and is often protected by specific legal devices [29,44,46,55,64]. In contrast, non-participation or treatment refusal may stem from technical, professional, or personal reasons without an explicit ethical basis [19,56]. Authors warn that conflating these terms risks justifying negligent behaviors—such as patient abandonment—under the guise of conscience, or restricting legitimate dissent within professional practice [20,49,61].

Consideration of the above observations in our analysis led us to coin a new general definition for CO: *CO refers to a healthcare professional’s refusal to participate in a legally authorized procedure due to deeply held personal beliefs*. While some variations in wording exist, most previous definitions included three core elements: (a) refusal to participate, (b) in a lawful healthcare service, (c) based on reasons of conscience. However, interpretations of these elements vary across the literature, as described below.

**(a) Refusal to participate**. Interpretations of refusal range from positive framings (e.g., beneficent refusal, ethical refusal) [19,65] to more critical ones (e.g., convenience objection, conscience creep, treatment deniers, unconscientious objectors, or dishonorable disobedience) [20,41,49,56,60,61]. Proponents of CO do not regard the refusal to participate a privilege [59], or a moral courtesy [52]; rather, they consider it a fundamental right of healthcare providers [24,46,54,59]. Why? CO is rooted in freedom of religion and conscience, which are broadly recognized human rights [24,30,43,48,69]. However, these rights are not absolute and may be restricted to protect equally fundamental rights, such as access to equitable care and the right to die with dignity [20,21,25,34,36,46,49,50,52,59,69,72]. A legal analysis is therefore necessary to translate this ethical issue into sound public policy [37].

**(b) In a lawful healthcare service**. Several articles adopted a technical, legislative stance, differentiating between *positive* and *negative* rights [26,32,35,65]; *privilege* and *claim* rights [27,28]; rights *in rem* or *in personam* [21,37]; *qua external* and *qua internal* [37]. The results of these articles’ analyses highlighted that the situation varies across countries and jurisdictions. In the Netherlands, for example, patients have the right to request EAS but cannot demand it from any specific physician [28]. In contrast, in Spain, EAS is considered to be a patient’s subjective right, obligating state provision [30].

**(c) Based on reasons of conscience**. The reasons for invoking CO can be broadly categorized into three types: religious beliefs, moral/secular reasons, and emotional/psychological motivations. While these categories differ, authors highlight that they are all closely linked to personal identity, which makes them deserving of respect [21,32,42,49,55,63,65,70]. Disregarding these motivations may have substantial effects on the individual, who may perceive it as a form of self-betrayal [34,41,72]. Regarding religious beliefs, authors defending their legitimacy often reframe them in publicly reasonable terms, such as natural law, care for the vulnerable, and human dignity [24,32,33,66,76]. Critics argue that religious motivations are inherently personal and arbitrary, making it difficult to distinguish legitimate claims from self-interested or unjust ones, thus rendering CO practically untenable [21,32,61,62,68,75]. Moral or secular reasons, on the other hand, include philosophical principles that are not necessarily tied to a revealed tradition. Non-religious objections often refer to conflicts with the Hippocratic Oath [24,29,33,36,55,63,66,76]; medical ethics codes [24,50,63,65]; traditional goals of medicine [24,33,41,54,55,65,72]; and foundational principles of the law [50,54,69]. Finally, psychological motivations are often invoked to describe the personal harm and emotional distress that violating one’s conscience can inflict on the individual’s well-being [21,32,34,39,43,55,56].

### *Who* can object to EAS practices?

The question of who may be entitled to object remains hotly debated. In our analysis, 29 articles focused on CO in relation to physicians, while 3 focused on nurses, and 3 focused on pharmacists. The remaining 23 articles were more broadly inclusive, using terminology that covered all clinical healthcare professionals.

Defining entitlement for CO requires first clarifying which specific actions can be objected to, such as providing EAS information, handling requests and assessments, prescribing or administering medication, and/or being present during administration [36,42,47]. Some authors advocate for a dichotomous characterization, distinguishing between activities that are “hands-on” *versus* “hands-off” to differentiate direct from indirect participation; this characterization helps to define who may legitimately object [34,40,49].

Articles on CO that focused on nurses and pharmacists often emphasized their direct involvement in the procedure [19,40,45,51,53,57]. They are moral agents and not mere executors of the physician’s orders. Their concerns point to legal vulnerability and the potential harm to patients [19,45,49,53,57]. A notable case among the articles we analyzed was that of CO of junior doctors, who feel particularly vulnerable in asserting CO rights due to their subordinate role in medical hierarchies and reliance on senior physicians for training and career progression [47].

### *Which* objections are acceptable?

Despite the long-standing tradition of CO, its role in contemporary medicine remains contentious, particularly within publicly funded healthcare systems [20,24,32,38,50,54,65]. Even advocates of CO acknowledge that not every appeal to conscience should be automatically granted [21,33,50,68], as this could lead to arbitrary healthcare provision and discrimination [56,57,59,64,66,69,72]. Several scholars caution against the potential for misuse of CO [38,42,46,61]. Establishing acceptance criteria for CO is therefore crucial. In this context, defenders of CO seek ethical justifications for each of the motivational drivers behind conscience-based refusals. Advocates for religious beliefs base their arguments on the fundamental right to freedom of conscience and religion [24,31,43,54,69], while those supporting moral and secular reasons emphasize their capacity to withstand public scrutiny [32,42,52,67,69]. Proponents of psychological and emotional motivations highlight the potential long-term negative consequences for the individual, such as moral distress and burnout [34,38,45,47,53,63,71].To navigate this complexity, some authors have developed criteria to assess the *genuineness* and *sincerity* of objections, emphasizing their consistency with prior values and substantial commitments [31,41,56,68,70]. Nevertheless, applying these criteria remains challenging [42,51,61].

Other authors propose *reasonableness* criteria, grounded in rationality and communicability [41,50,70], often drawing from philosophical frameworks such as Rawls’ concept of public reason [26,32,42,58,68]. In a monograph, Reichlin outlined four external and four internal conditions necessary for valid objections [58]. However, Zolf analyzed and rejected these arguments, concluding that such criteria were too ambiguous and lacked normative justification [75].

Authors like Pellegrino [55], Wicclair [72], and Sulmasy [64] supported CO based on *extrinsic* justifications, such as the value of tolerance and diversity in democratic societies, weighed against an acceptable level of harm to the patient. To prevent CO from enabling discrimination, some authors suggested limiting objections to specific acts rather than particular individuals [32,42], or permitting objections only if aligned with the goals of medicine [20,40,42]. However, these proposals also failed to achieve unanimous consensus [73,75].

### *Why* is CO ethically contentious?

Our results show that CO has received increased academic attention following the legalization of EAS in countries like Canada, Spain, and Australia. Several authors discussed worldwide *societal changes* as a factor contributing to this increased contention [21,29,44,54,55,69,74]. For instance, a growing emphasis on patient autonomy may partially explain the rising demand for EAS and, thus, the questioning of CO [30,31,40,43,58,68,72]. Some viewed the emphasis on personal autonomy as a cultural achievement [26,39,41,46,53,60,69,74], while others viewed it as a form of tyranny, raising concerns about the risks of excessive individualism [21,25,43,45,48,50,54,55,57,67].

Another factor contributing to contention in the academic dialogue about CO is authors’ tendency toward *oppositional* framing in their discussions. Some articles presented CO as a *conflict* between mutually exclusive rights [20,24,27,34,36,56,69,70]; some described CO as a *clash* between personal views [20,36,41,68,72]; others portrayed CO as a *dilemma* between conflicting values, duties, or principles [19,26,27,32,39,53,55,58,59]. Some authors highlighted the power imbalance between healthcare providers and patients, with the latter being assigned as the vulnerable party [34,39,42,46,48,70].

At times, this oppositional thinking could lead to *confrontational* language in their discussions [25,44,50,52,71], with some using phrases like “cultural wars” or “weaponization” of medicine [48,61,64,69]. Nevertheless, several authors emphasized that the conflict is not primarily external—where the healthcare provider is pitted against the patient—but rather, it is internal, with healthcare providers torn between their convictions to carry out their duties toward themselves and their patient [19,22,27,31,45,57].

### *How* is CO applied in EAS?

Several articles begin by providing a historical overview of CO, tracing its origins in military service and its expansion into healthcare, initially through abortion and contraception, and more recently, in end-of-life care [34,50,61,69,71]. This historical context is crucial for understanding how CO has evolved and how it is applied in modern healthcare settings. The analyzed publications did not limit CO to abstract theoretical discussions; instead, they used case studies to analyze and offer practical solutions [22,39,53]. They emphasized the *complexity* of real-world situations, challenging simplistic interpretations of CO [21,41,47,57,69,70]. Several articles advocated for a context-sensitive, case-by-case approach to CO in EAS, which better reflects the lived realities of healthcare professionals and allows for navigating the moral “gray zones” that often arise in end-of-life care [21–23,40,57,72,73].

Authors also explored the consequences of CO for various *stakeholders.* Some articles focused on the harm caused by CO to *patients*, particularly regarding delays and possible harm caused to denying equal access to healthcare [20,24,34,38,41,42,47,52,53,56,62,65,75]. Others discussed the impact CO could have on *colleagues and institutions*, such as increased workload and moral decline [19,35,38,51,52,61,62,70]. Finally, some authors addressed the potential consequences *healthcare professionals* face if CO is not permitted [51,60,62,66]. In such a situation, objectors might even have to consider leaving the profession, switching to specialties where these issues are less likely to arise, or abandoning their conscience altogether [20,24,48,49,55,60,61,71,76].

Conflicts between stakeholders were frequently addressed through policies and practical solutions within specific *healthcare settings and legislation* [37,38,41,42,51]. Analysis of the first author’s affiliation revealed that 14 different countries were represented. A notable concentration of first author affiliations were in countries with EAS legislation (cf. Table 3). Furthermore, a thorough examination showed that all countries where EAS is currently legal are discussed in the reviewed articles. Authors highlighted that CO jurisdictions vary widely. While the USA was cited as an example country where CO is strongly protected by legislation [34,37,71], Sweden and Finland are examples of countries where CO is not recognized within healthcare [34,44,60]. Between these two extremes, most countries adopted a “compromise approach,” aiming to accommodate CO while also imposing certain limitations [36,48,54,57,59,63,68].

### Ethical positions on and main arguments of CO

Our second research question aimed to identify the main ethical positions and related arguments of CO in EAS. Our analysis indicated that researchers broadly agree on using the threefold categorization established by Wicclair, albeit with minor terminological variations. [5]

At one end of the spectrum was *conscience absolutism.* This position advocates for the absolute protection of healthcare providers’ conscience and supports their right to refuse any participation in EAS. Among the key proponents of conscience absolutism is Pellegrino, who calls for full protection of CO, while at the same time, urging doctors not to abandon their patients and to communicate their objections “courteously but definitively” [55].

At the opposite end of the spectrum was the *incompatibility thesis.* This position argues that CO is incompatible with healthcare professionals’ duties and that they should provide all services prescribed by law, regardless of their personal beliefs or convictions. Savulescu and Schuklenk are two prominent authors that support this stance [60–62].

Between these two extremes lies the *compromise approach*. This position seeks to find a middle ethical ground by accommodating CO but imposing certain limitations. The *compromise approach* aims to balance the rights of both healthcare providers and patients. Wicclair, the most influential author associated with this perspective, bases his position on respect for professional moral integrity [72].

Arguments for and against each ethical position are presented in Table 4.

**Table 4:** Arguments for and against the three main ethical positions on CO in EAS.

	ARGUMENTS FOR	ARGUMENTS AGAINST
Conscience absolutism	✓ Protects fundamental human rights: freedom of religion and conscience ✓ Defends the moral integrity of the individual, both professional and personal ✓ Acknowledges the controversial moral status of intentional killing ✓ Prevents moral complicity in controversial actions ✓ Concerns for vulnerable patients and the moral integrity of the profession ✓ Upholds traditional medical values, such as those in the Hippocratic oath ✓ Highlights the role of conscience in medical practice and clinical judgement ✓ Protects healthcare professionals from external interferences References: [29–31,41,43,44,52,55,63,65,67,69,71]^a^	⊗ Prioritizing individual conscience over the rule of the law risks societal harm ⊗ Neglecting other values like patient autonomy fosters paternalism ⊗ Power imbalance between physicians and patients demands protection ⊗ Untestable conscience claims allow arbitrary and discriminatory practices ⊗ Professional duties to ensure fair/ equal care should outweigh personal beliefs ⊗ State neutrality prevents favoritism in democratic societies ⊗ Objections should be discussed before laws are enacted not during care References: [20,34,35,39,41,42,46,48,58–60,63,70,74,75]^a^
Compromise approach	✓ Protects both patients’ and professionals’ rights ✓ Respects CO but sets reasonable limits and conditions ✓ Promotes a nuanced, context-sensitive, case-by-case approach ✓ Balances respect for patients’ autonomy and practitioners’ moral integrity ✓ Defends tolerance and moral diversity in pluralistic societies ✓ Encourages ethical reflection and epistemic humility regarding moral issues References: [19,21,24,31–33,41–43,49,50,52,56,64,66,68,69,71,72]^a^	⊗ Accommodation may be an unsatisfactory compromise for both sides ⊗ Objectors risk feeling moral complicity in EAS practices ⊗ Accommodation poses unsolvable practical and logistical challenges ⊗ Burdens patients, colleagues, and institutions ⊗ Patients face delays and impediments, especially in rural and remote areas ⊗ CO accommodation creates inequity and inefficiency in patient care References: [20,24,27,28,35,48,54,60–63,68,73]^a^
Incompatibility thesis	✓ Emphasis on professional obligations and fiduciary duties ✓ Patients’ interests take priority over personal beliefs ✓ Healthcare professionals are public servants and monopoly providers ✓ Healthcare professionals must ensure equal access to legal treatment as part of a social contract ✓ Prevents discrimination of vulnerable populations and remote areas ✓ Professionals voluntarily choose and must adapt to evolving medical practices ✓ Objectors can seek alternatives, like changing specialty or practicing field References: [20,21,24,26,35,49,56,60–62,71,74,75]^a^	⊗ Forcing physicians to act against their beliefs puts them at risk for moral distress and burnout ⊗ Physicians are not blind executors; they are moral agents that deserve respect ⊗ Potential discrimination toward conscientious objectors ⊗ Risk of shortages in certain specialties or in underserved areas ⊗ This thesis undermines moral diversity and pluralism in the medical profession ⊗ State-imposed moral decisions risks totalitarianism ⊗ Legislation may be fallible; patient autonomy is not medicine’s only goal ⊗ Less restrictive alternatives can achieve the same results References: [19,20,28,29,34,43,54,55,58,59,63,65–68,71,72,76]^a^

Abbreviations: CO, conscientious objection; EAS, euthanasia and assisted suicide.

^a^References cited in this table illustrate an ethical position without implying the authors’ personal endorsement.

Even though most authors refrained from extreme positions and advocated moderate approaches, reaching a compromise that satisfies all parties remains challenging. Across the reviewed articles, four key conditions for CO emerged as central to the debate. These are considered in turn.

### Effective referral

The first condition involved ensuring effective referrals. In many jurisdictions, objectors are required to refer patients to healthcare professionals willing to provide EAS (i.e., an effective referral) to ensure patients’ timely access to lawful care and prevent abandonment [22,23,31,32,39,40,45,48,53]. However, some authors expressed discontent with this policy, arguing that an effective referral constitutes a form of participation that is morally indistinguishable from directly performing the referred intervention [33,48,73,76]. The Canadian court acknowledges that such a policy infringes, in a nontrivial manner, on the doctor’s right to freedom of conscience; yet justifies this infringement in light of the state’s interest in ensuring equitable access to healthcare [20,34,43,48]. Some authors have gone further, suggesting that objecting physicians could be compelled to perform euthanasia themselves when an effective referral is not possible — for instance, in life-threatening emergencies (though unlikely in the context of an EAS request) or in very remote areas where no alternative providers are available [21,28,31,32,41,48,68,71].

### Obligation to inform

The second condition that emerged as central to the debate involved ensuring an obligation to inform. This issue is particularly debated in certain jurisdictions of the USA and Australia, where healthcare providers are legally required to inform patients about the possibility of EAS as an end-of-life option [23,36]. However, different interpretations of the law exist regarding whether there is an affirmative duty to actively inform or simply respond to patients’ requests for information. This raises key questions about who should initiate the conversation, what information should be provided, and how it should be communicated in a sensitive manner [23,32,36,41,46]. In this context, some authors argue that CO must be counterbalanced by patients’ rights to information, equal access to care, and non-discrimination, compelling doctors to fulfill a ‘facilitating obligation’ as a means of balancing competing rights [28,36,39,59,68,71]. The debate in these jurisdictions centers on whether healthcare providers should initiate the conversation about EAS or wait for the patient to raise the issue. Critics of proactive information fear it could be coercive, while supporters argue it ensures patients are aware of their legal rights and options [23,36].

### Advance notification

The third condition that emerged as central to the debate involved ensuring advance notification. To minimize the burden on patients, colleagues, and healthcare institutions, objectors may be required to notify others about their objection to EAS in advance; that is, before a request for EAS is made. This should be carried out in a timely, sensitive, and respectful manner, avoiding any moral judgment about the patient [31,41,42,55,56,63,69,71].

### Registration of professionals

The last condition that emerged as central to the debate involved ensuring registration of healthcare professionals. Some jurisdictions have compiled a “register of objectors” as a mechanism to introduce a degree of public scrutiny [42,59]. However, critics raised concerns about privacy, confidentiality, and potential employment consequences for objectors [30,59,63,76]. Alternatives include “registers of providers” or a centralized care coordination system, as implemented in New Zealand and some Canadian provinces [34,38,48,59,76]. These mechanisms aim to empower patients by facilitating direct contact with providers willing to carry out EAS [38,39,63].

### Underlying presuppositions of CO

The ethical positions on CO and related policies in our reviewed literature—whether aimed at protecting, accommodating, or rejecting the practice—have been presented. While these positions have now been clarified, the debate moving forward on whether CO in EAS safeguards professionals’ moral integrity or obstructs patients’ access to care may remain stalled until the often implicit underlying presuppositions are critically examined.

Our third research question aimed to identify the underlying presuppositions that shape the debate on CO in EAS. To address this issue, we now highlight the foundational concepts that emerged across the analyzed publications.

### The nature of conscience

Some authors trace the concept of conscience back through its long history in philosophical thought [45,55,63,70]. According to classic definitions, they describe it as the human capacity to discern good from evil [43,45,55,56,63,64,67,68]. Authors analyzed conscience from different perspectives, focusing on its rational, emotional, intuitive, or practical dimensions [19,22,32,33,41–43,45,55,56,63–65]. Traditionally, in the arena of moral decision-making, conscience has been granted a special status: Individuals must follow their conscience and should not be forced to act against it [21,32,48,55,64,70]. While traditional views of conscience often portray it as static and individualistic, alternative approaches have emerged, rooted in personalist and relational grounds [22,25,35,38,45]. These alternative approaches challenge the idea of conscience as an opaque, untestable “black box” [38,41]. Given the diversity in how conscience is interpreted, some authors argued that regulating conscience is practically unfeasible [19,21,27,32,60–62,66,68,75].

### Moral integrity and complicity

An analysis of conscience revealed a close connection to moral agency and personal identity [21,22,31,32,41,42,47,49,55,63,65,70,72]. Acting against one’s conscience constituted a form of self-betrayal or self-harm [19,34,38,41–43,71,72]. Consequently, an attack on conscience is considered to be an affront to human dignity and moral integrity [25,42–44,50,54,69,72]. The consequences of violating one’s conscience ranged from psychological harm—such as feelings of guilt and remorse—to moral distress, emotional exhaustion, and professional burnout [21,22,24,38,39,41,43,45,46,53,63,70,71].

Moral complicity in perceived immoral actions was another recurring theme in the reviewed literature, with 32 out of 58 articles addressing it. It was frequently discussed in relation to controversial procedures such as prescribing EAS medication, providing mandatory referrals, giving advance notification, or fulfilling the obligation to inform [19,28,34,36,39–41,58,68,71–73,76]. In this regard, various authors provided insights into evaluating the degree of moral complicity and determining the legitimacy of different COs [22,24,27,32,40,41,48,58,68,71,73].

### The role of religion in CO

While reasons for CO are not necessarily religious in nature, many authors highlighted the importance of religious-based arguments [22,23,27,29,32,34,43,52,57,60,73]. Some authors were particularly critical of this viewpoint, while others defended its validity [21,24,28,31,33,36,49,62,66,75]. It is often argued that public institutions, like the state and courts, should maintain neutrality when it comes to invoking religious reasons for CO [20,36,43,46,60,67].

Underlying this debate are contrasting views on the role of religion in civil society. Several authors described this phenomenon in terms of secularization [20,21,52,54,55,60,67]. Some warned that extreme secularism could lead to intolerance, pointing to the potential discrimination that religious professionals could face due to their beliefs as it relates to CO in EAS [19,29,43,54,55,68,71,76].

### The transformation of medicine

Our analysis of included articles described how medicine is undergoing a transformation process, moving from its traditional focus on health and the preservation of life to a more business-like focus in delivering healthcare [29,33,44,50,55,63,66,68]. Emerging perspectives had redefined medicine as the provision of goods and services, prioritizing client rights [26,36,38,61,68,74]. Indeed, authors viewed medicine as evolving from a vocational profession to a consumer service based on individual choice [28,29,52,55,63,65].

In this context, various authors defended the role of conscience in contemporary medicine [21,22,25,33,42,45,55,64,65]. Sulmasy, for example, advocated that conscience is essential to good medical practice, and is not limited to extraordinary moral dilemmas; engaging one’s conscience, he argued, is integral to proper clinical judgment [64]. In contrast, Schuklenk contended that patients seek physicians for their technical expertise not for their moral guidance, rendering professionals’ personal views and convictions as irrelevant to the doctor-patient relationship [62].

### End-of-life ethical considerations

Although the moral judgement of EAS was not the primary focus of the articles we analyzed, its ethical appraisal deeply influences the justification of CO as a legal exception. Authors argued that the legitimacy of CO depended on how these practices were framed: as a standard medical procedure [60,62,76]; a subjective right of the patient [30,54]; an intrinsically evil action [68,73]; or an act contrary to the goals of medicine [24,59,63]. Despite these differing views, the controversial nature of EAS was widely recognized, as these practices involved situations of *moral gravitas* [45], for which there was no moral consensus [19,37,43,64,66,68,69,74]. In this regard, several authors cautioned that the “intentional taking of human life” demands exceptional justification and should not be trivialized, highlighting the relevance of CO in end-of-life care [32,52].

## Discussion

Healthcare professionals increasingly face ethical dilemmas regarding their participation in EAS, as some perceive these practices to conflict with their deeply held moral convictions. However, the polarized debate about those declining to participate (e.g., CO) has been somewhat muddied to date. To clarify key issues, we systematically analyzed the argument-based literature on CO in EAS, addressing the research questions on the meaning and uses of CO among healthcare professionals, the main ethical positions and arguments, and the underlying presuppositions shaping the debate. Following our analysis, this extensive body of literature on CO can now be situated more firmly within the broader context of existing research exploring the following themes: (1) language and framing of CO, (2) different levels of discussion about CO, (3) contextual factors influencing CO, and (4) ongoing debates on CO. Our review and analysis show that clearer guidelines are needed to balance respect for conscience, patient rights, and professional responsibilities.

### Linguistic and legal framing of CO

Our findings highlight the need to distinguish CO from related phenomena—such as refusal to treat, non-participation, patient abandonment, and professional dissent— and underscore the importance of differentiating these concepts in light of relevant literature. While CO is typically grounded in moral or religious convictions, Martins-Vale et al. note that ‘refusal to treat’ may arise from other legal, technical, or personal motivations [6]. Similarly, Brown emphasizes the need to differentiate CO from ‘non-participation’, which may be driven by professional ethos, emotional labour, or systemic constraints [77]. Other authors caution that ‘patient abandonment’ can occur if CO leads to failure in ensuring continuity of care, raising concerns of professional misconduct [78,79]. Finally, ‘professional dissent’ may be the appropriate term when objections are based on principles and values intrinsic to the healthcare profession, rather than in personal moral beliefs [80]. Clarifying these distinctions is essential for accurate ethical and legal interpretation.

In addition, our study highlights the importance of language in shaping how CO is framed and interpreted. While CO is the predominant term, variations in terminology exist and deserve consideration. Jones-Nosacek, for instance, argues that “conscientious objection” is a more accurate term than “conscientious refusal,” as objectors are not rejecting care but are acting on moral convictions [81]. On the other hand, Strouse critiques CO as a “toxic form of patient abandonment” [82], a view echoed by authors in our analysis who label objectors as “treatment deniers” or “unconscientious objectors,” implying deliberate obstruction of access to legally sanctioned healthcare [41,60].

As our findings pointed out, the justification for CO largely depends on the *legal framing* of EAS [26,35,37]. One approach considered EAS to be a negative claim right *in rem*, where patients have the right to request EAS, but their request does not need to be granted by a given physician [28,39,54]. Another approach considered EAS to be a positive claim *in rem* and *in personam*, implying that doctors have a fiduciary duty to patients—due to the vast asymmetry between them—and that the state commits to ensuring access by facilitating its funding and including it in the service portfolio [30,69]. Each approach generates distinct rights and obligations on the side of healthcare professionals. Our findings highlighted the lack of agreement in interpreting often ambiguous legislation, a point regularly affirmed in the literature [2,4,7,82]. Clarifying the language and framing will help to close this gap.

### Two levels of discussion about CO

Our analysis revealed *two distinct levels of discussion* regarding CO: the pragmatic level, which focuses on procedures for implementing CO in EAS; and the foundational level, which focuses on the underlying premises. The complex relationship between theoretical models of conscience and practical approaches to medicine is also explored in the broader literature [83,84]. Building upon this distinction, a significant body of *empirical research* on CO in EAS reveals a notable gap between theoretical models and clinical practice. While theoretical frameworks often presented CO as a binary choice (i.e., either to object or to participate based on fixed ethical principles) [22,57], empirical studies emphasize the role of contextual factors in healthcare professionals’ decisions to participate in EAS [85,86]. These factors include external elements such as institutional policies, logistical barriers, and community norms [87,88], in addition to internal elements like professional experiences and emotional considerations [87,89]. Some studies suggest that refusals of professionals to participate may arise more from practical challenges, such as time constraints or legislative uncertainties, than from moral opposition [88,90]. These socially mediated and situational refusals blur the distinction between genuine CO and other forms of refusal or dissent. As a result, the complexity of CO in practice is often oversimplified in policy and legal frameworks, as many healthcare professionals adopt fluid, context-dependent positions rather than rigid moral stances [7,85,89]. This gap is addressed in our review through a call for greater conceptual clarity and a theoretical framework that incorporates contextual sensitivity and a case-by-case approach to defining and regulating CO. [21–23,41,53,57,59,72,73].

Studies on CO confirm that context plays a crucial role in shaping refusals to provide care, influenced by factors such as institutional culture, peer expectations, or sociocultural norms. Harris et al. suggest that CO may serve as a strategy to avoid social stigma associated with controversial medical procedures, specially under challenging working conditions [91]. Bouthillier similarly suggests that some healthcare professionals may invoke CO to conceal emotional or professional vulnerabilities, rather than expressing genuine moral beliefs [86]. Lamb warns against conflating CO with technical, administrative, or emotional refusals, a confusion that undermines its ethical and regulatory clarity [92]. Building on these insights, Sedgwick proposes a conceptual distinction between objections *to* EAS and non-participation *in* EAS [85]. These studies highlight the need for greater conceptual clarity in the discourse on CO, urging a more nuanced and contextually informed framework for its ethical and legal interpretation.

### Contextual factors influencing CO

An interesting point of discussion is whether the ethical considerations of CO differ when applied to *EAS compared to other practices* where complex ethical decisions are made, like abortion. First, some authors emphasize the unique circumstances of end-of-life decisions. EAS typically does not involve emergency situations where a patient’s prognosis may change for the better. Additionally, the patient often lacks the ability to relocate to a different healthcare facility. Therefore, policies governing CO in abortion may not generalize to EAS [93,94]. Second, EAS is regulated by more recent legislation, and with societal views still evolving, this may explain the higher rate of CO in EAS at this time [94]. In the same vein, one of our reviewed articles suggested that rapid societal changes will make CO in EAS as contested as refusals of reproductive treatments are today [60].

Another contextual factor explored in the review that influences CO is *the role of religion* in healthcare. Weinstock questions whether religion still plays a significant role in conscientious refusals among healthcare professionals [95], while Magelssen argues that as secular attitudes in society become more prevalent, support for CO may decline [96]. This raises doubts about whether current policies tolerating CO in practice will remain acceptable to the majority [2,20,60,62,75,84].

### Ongoing debates on CO

While our review focused primarily on individual CO, broader debates about its application extend beyond this scope. One such debate, the *asymmetry thesis,* questions whether CO should apply not only to those who refuse participation in EAS but also to those who wish to provide it in jurisdictions where it is prohibited [4,97,98]. Another discussion concerns *institutional objections.* Some authors argue that institutions should be allowed to object to EAS in order to preserve their established identity, while others contend that conscience is solely an individual attribute [5,99–101].

Finally, another open debate involves entitlement: Who has the right to object? Although our review focused on healthcare professionals, the ethical literature also considers objections from *non-clinical staff,* such as professional interpreters [102], and educators teaching end-of-life care [103]. These ongoing discussions underscore the complexity and evolving nature of CO in the context of EAS.

### Strengths and limitations

We acknowledge certain limitations of our review. Despite the inclusion of diverse sources from around the world, our exclusive use of English-language search terms may have omitted perspectives from non-English-speaking regions (cf. Table 3). Our inclusion and exclusion criteria, while designed to ensure conceptual clarity and avoid conflating different debates, may have excluded valuable contributions. By focusing on argument-based literature and limiting our scope to healthcare professionals, we may have narrowed the range of perspectives captured. If we had included articles on CO related to institutions and non-clinical staff, we might have captured more perspectives, but at the risk of diluting the focus on patient care. Thus, our more focused approach aimed to maintain a clear light on individual CO in direct patient care.

Despite these limitations, the review has notable strengths. The search strategy was particularly rigorous and comprehensive, involving 13 databases and employing validated techniques. This approach identified a substantially larger body of literature compared to previous similarly themed systematic reviews [6–8]. The results are systematically presented in direct response to the clear research questions, offering a well-structured analysis that clarifies key themes and arguments. The predominance of recent publications underscores the growing relevance of the topic, and the saturation of findings suggests a comprehensive capture of primary discussions. Hence, we believe that this review will provide a reliable resource for healthcare professionals, policymakers, and scholars.

## Conclusions

As more countries consider legalizing EAS, the issue of CO is becoming increasingly important for healthcare professionals and policymakers. Our analysis leads to several conclusions. First, the debate on CO is often polarized and framed as a conflict between healthcare professionals and patients. However, a deeper examination of the ethical arguments in the literature suggests that healthcare professionals are concerned not only with safeguarding their own conscience but also with respecting patient autonomy. Similarly, patients may prefer care from professionals who uphold strong moral standards. Thus, both parties share a fundamental interest in fostering mutually respectful solutions.

Second, discussions about CO typically occur at two levels: one pragmatic and procedural, the other theoretical and fundamental. While substantial literature exists on both levels, the lack of dialogue between them may hinder the effective implementation of CO in EAS. Therefore, fostering dialogue between these levels, and among various stakeholders, is crucial. Simple or definitive solutions are unlikely, and navigating the complexities of real-life clinical practice is essential.

While CO remains a central issue in healthcare ethics, clearer guidelines are urgently needed for its proper application, particularly in end-of-life care. This requires bridging theoretical insights with the lived experiences of healthcare professionals and patients. Further research is necessary to strike a balance between respecting conscience, safeguarding patient rights, and fulfilling professional responsibilities.

## Supporting information

S1 File PROSPERO registration. (PDF)

S2 File PRISMA 2020 Checklist. (PDF)

S3 File RESERVE Checklist. (PDF)

S4 File TARCiS Checklist. (PDF)

S5 File Full search strategy. (PDF)

S6 File Example of conceptual scheme. (PDF)
