# Supplementary material for: Conscientious objection in euthanasia and assisted suicide: A systematic review
Source: PLoS One. 2025 Jun 23;20(6):e0326142. doi: 10.1371/journal.pone.0326142 (PMC12185019; doi:10.1371/journal.pone.0326142)
Supplement: S1 File — (PDF) [file pone.0326142.s001.pdf]

NIHR | National Institute for Health and Care Research

PROSPERO
International prospective register of systematic reviews

Conscientious Objection in Euthanasia and Assisted Suicide: a Systematic Review of Argument-Based Literature

Carlos Gómez-Vírseda, Chris Gastmans

To enable PROSPERO to focus on COVID-19 submissions, this registration record has undergone basic automated checks for eligibility and is published exactly as submitted. PROSPERO has never provided peer review, and usual checking by the PROSPERO team does not endorse content. Therefore, automatically published records should be treated as any other PROSPERO registration. Further detail is provided here.

Citation 1 change
Carlos Gómez-Vírseda, Chris Gastmans. Conscientious Objection in Euthanasia and Assisted Suicide: a Systematic Review of Argument-Based Literature. PROSPERO 2024 CRD42024592004. Available from https://www.crd.york.ac.uk/PROSPERO/view/CRD42024592004.

REVIEW TITLE AND BASIC DETAILS

Review title
Conscientious Objection in Euthanasia and Assisted Suicide: a Systematic Review of Argument-Based Literature

Review objectives 1 change
What does Conscientious Objection mean and how is it used in argument-based literature on Euthanasia and Assisted Suicide (EAS)? What are the arguments used for and against the practice of Conscientious Objection in Euthanasia and Assisted Suicide? What are the underlying presuppositions that shape the debate on Conscientious Objection in Euthanasia and Assisted Suicide?

Keywords
Assisted Suicide, Bioethics, Conscientious Objection, End-Of-Life Care, Euthanasia

SEARCHING AND SCREENING

Searches 1 change

A systematic review of argument-based ethics literature will be conducted in the following databases: PubMed, Embase, Web of Science, CINAHL, SciELO, Scopus, ProQuest Central, Philosopher's Index, JSTOR, Phil Papers, ATLA, Index Religiosus, and Index Theologicus. Starting on September 18th 2024, the search string consist of two categories of words: A) conscientious objection and B) Euthanasia and Assisted Suicide. All databases will be queried using Boolean searches expressed in English. Additional relevant publications will be identified via forward and backward citation searching, following TARCiS recommendations.There will be no time or language restrictions. The systematic review process will adhere to the RESERVE (Kahrass et al. 2023) and PRISMA (Page et al. 2021) guidelines.

Study design 1 change

Both randomized and nonrandomized study types will be included.

Included

Only argument-based literature will be eligible, meaning works that apply ethical concepts, derived from current or traditional theories, to address conceptual issues and argue for a specific position or conclusion. Articles must meet the following inclusion criteria: (1) Focus on individual conscientious objections of any clinical healthcare professional, (2) Substantially applied to euthanasia or assisted suicide (EAS) practices,(3) belong to argument-based literature, (4) no time restriction, and (5) no language restrictions.

Excluded

Non-peer-reviewed articles; non-academic sources (e.g., blogs or magazines); conference abstracts; preprints; book chapters or dissertations. Empirical studies; opinion pieces (e.g., editorials); literature reviews; technical reports; guidelines; protocols, ethics policies, or ethics codes. Focus on non-clinical health professionals or institutional conscientious objections. Insufficient or unclear application to EAS practices.

Link to search strategy 1 change

A full search strategy has been uploaded to PROSPERO. The PDF may be accessed through this link https://www.crd.york.ac.uk/PROSPERO/PROSPEROFILES/36bd375057ef1e73be0640724c6f2825.pdf.

ELIGIBILITY CRITERIA

Condition or domain being studied 1 change

Conscientious objection refers to an individual's refusal to fulfill a legal duty based on ethical or religious convictions. In the context of end-of-life care, this means healthcare professionals may refuse to provide specific services, such as Euthanasia or Assisted Suicide (EAS), due to conflicts with their personal conscience. While conscientious objection is a well-established practice in medicine with deep historical roots, its practical application in real-world settings presents challenges for healthcare providers, their colleagues, and the patients. Therefore, a study that clarifies the use of conscientious objection in this context is essential to enhance its implementation in end-of-life care, ensuring it is carried out respectfully for all stakeholders involved.

Population

Since we are conducting an argument-based systematic review, this specific question is not applicable to our research.

Intervention(s) or exposure(s)

Since we are conducting an argument-based systematic review, this specific question is not applicable to our research.

Comparator(s) or control(s)

Since we are conducting an argument-based systematic review, this specific question is not applicable to our research.

OUTCOMES TO BE ANALYSED

Main outcomes

The main outcome of this study is to identify and analyze the ethical arguments both for and against conscientious objections to Euthanasia and Assisted Suicide, as well as the intermediate positions that advocate for accommodation.

Measures of effect
Not applicable

Additional outcomes

Not applicable

DATA COLLECTION PROCESS

Data extraction (selection and coding)

Articles will be selected based on previously defined inclusion and exclusion criteria (see above). To be included in the systematic review, candidate articles have to meet all inclusion criteria and have no exclusionary one. Title and abstract screening will be performed independently by two authors. Both appraisers will discuss every doubtful candidate article individually until consensus will be reached.
Since there is no standard for the quality appraisal of argument-based literature, we will rely on the peer review process and the academic reputation of the journals to ensure the quality of the included studies.
Data extraction and analysis of the results will follow the five procedural steps outlined in the Qualitative Analysis Guide of Leuven -QUAGOL- (De Casterlé et al. 2012). First, the articles will be read and reread, with key sections highlighted. Second, a narrative summary will be developed to outline the main arguments and identify core concepts. Third, a conceptual scheme will be created for each publication, presenting and interrelating relevant concepts to address the research question. Fourth, these individual schemes will be integrated to reveal relationships and provide a comprehensive response to the research questions, with a global scheme constructed and iteratively checked for consistency. Fifth, the results will be synthesized and reported for the Results section of the review.

Risk of bias (quality) assessment 1 change

Two researchers will perform the title and abstract screening of identified articles in order to determine which meet the inclusion criteria. Any disagreement will be resolved through discussion until a consensus is reached. This approach will also be used for the evaluation of conceptual schemes.

Given the absence of an established standard for quality appraisal of argument-based literature, we adopted Mertz's strategy of "appraisal using procedural quality assurance criteria" (Mertz, BMC Medical Ethics, 2019). Hence, we relied on the peer review process and the academic publisher's reputation to ensure the adequacy of the included publications.

PLANNED DATA SYNTHESIS

Strategy for data synthesis

We will adhere to the five successive steps of the analysis process outlined in Qualitative Analysis Guide of Leuven QUAGOL (Dierckx de Casterlé et al., 2012) -see above-. This process will generate individual conceptual schemes for each article, which will then be integrated into an overall conceptual scheme addresing the research question. Data synthesis will involve iterative analysis within and across articles.

Analysis of subgroups or subsets

Not applicable

REVIEW AFFILIATION, FUNDING AND PEER REVIEW

Review team members

- Dr Carlos Gómez-Vírseda, KU Leuven
- Professor Chris Gastmans, KU Leuven

Review affiliation

KU Leuven

Funding source

Not applicable.

TIMELINE OF THE REVIEW

Review timeline

Start date: 18 September 2024. End date: 18 March 2025

Date of first submission to PROSPERO 1 change

18 September 2024

Date of registration in PROSPERO 1 change

30 September 2024

CURRENT REVIEW STAGE

Publication of review results

The intention is to publish the review once completed.The review will be publcished in English

Stage of the review at this submission 1 change

Review stage	Started	Completed
Pilot work	✓	✓
Formal searching/study identification	✓	✓
Screening search results against inclusion criteria	✓	✓
Data extraction or receipt of IPD	✓	✓
Risk of bias/quality assessment	✓	✓
Data synthesis	✓	✓

Review status
The review is completed.

ADDITIONAL INFORMATION

PROSPERO version history 1 change

- Version 2.0, published 27 Feb 2025
- Version 1.1, published 30 Sep 2024
- Version 1.0, published 30 Sep 2024

Review conflict of interest
None known

Country
Belgium

Medical Subject Headings
Dissent and Disputes; Euthanasia; Humans; Suicide, Assisted

Revision note 1 change
Updating the review at its current stage.

Disclaimer 1 change
The content of this record displays the information provided by the review team. PROSPERO does not peer review registration records or endorse their content.

PROSPERO accepts and posts the information provided in good faith; responsibility for record content rests with the review team. The guarantor for this record has affirmed that the information provided is truthful and that they understand that deliberate provision of inaccurate information may be construed as scientific misconduct.

PROSPERO does not accept any liability for the content provided in this record or for its use. Readers use the information provided in this record at their own risk.

Any enquiries about the record should be referred to the named review contact
