# Supplementary material for: Conscientious objection in euthanasia and assisted suicide: A systematic review
Source: PLoS One. 2025 Jun 23;20(6):e0326142. doi: 10.1371/journal.pone.0326142 (PMC12185019; doi:10.1371/journal.pone.0326142)
Supplement: S4 File — (PDF) [file pone.0326142.s004.pdf]

TARCiS Checklist for Terminology and Reporting of Citation Searching

**Terminology for Reporting of Citation Searching**	
RECOMMENDED TERM	DESCRIPTION
Citation Searching	Umbrella term.
Backward Citation Searching	Sub-method retrieving and screening cited references.
Reference List Checking	Sub-method retrieving and screening cited references by manually reviewing reference lists.
Forward Citation Searching,	Sub-method retrieving and screening citing references.
Co-cited Citation Searching	Sub-method retrieving and screening co-cited references.
Co-citing Citation Searching	Sub-method retrieving and screening co-citing references.
Iterative Citation Searching	One or more repetition(s) of a search method that exploits citation relationships.
Seed References	Relevant articles which are known beforehand and used as a starting point for any citation search.

**Reporting Item Checklist**			
SECTION/TOPIC	#	CHECKLIST ITEM	PAGE(S)
Methods			
Seed references	1	State the seed references (along with a justification should the seed references differ from the set of included records from the results of the primary database search).	8
Citation searching method	2	State the directionality of searching (backward, forward, co-cited, co-citing).	8
Search date	3	State the date(s) of searching (which may differ between rounds of iterative citation searching) (not applicable for reference list checking).	8-9
Number of iterations	4	State the number of citation searching iterations (and possibly the reason for stopping if the last iteration still retrieved additional eligible records).	9
Citation indexes and tools	5	State all citation indexes searched (e.g., Lens.org, Google Scholar, Scopus, citation indexes in Web of Science) and, if applicable, the tools that were used to access them (e.g., Publish or Perish, citationchaser).	Fig 1
Deduplication	6	State, if applicable, information about the deduplication process (e.g., manual/automated, the software or tool used).	Fig 1
Screening method	7	State the method of screening (i.e., state whether the records were screened in the same way as the primary search results or, if not, describe the alternative method used).	Fig 1
Results			
Search results	8	State the number of citation searching results in the right column box of the PRISMA 2020 flow diagram for new or updated systematic reviews which included searches of databases, registers and other sources.	Fig 1

From: Hirt J, Nordhausen T, Fuerst T, Ewald H, TARCiS study group, Appenzeller-Herzog C: The TARCiS statement: Guidance on terminology, application, and reporting of citation searching. January 2024.

Direct download of DOCX checklist: https://bit.ly/tarcisdocx
Direct download of PDF checklist: https://bit.ly/tarcispdf
