# Supplementary material for: Conscientious objection in euthanasia and assisted suicide: A systematic review
Source: PLoS One. 2025 Jun 23;20(6):e0326142. doi: 10.1371/journal.pone.0326142 (PMC12185019; doi:10.1371/journal.pone.0326142)
Supplement: S5 File — (PDF) [file pone.0326142.s005.pdf]

FULL SEARCH STRATEGY

GROUP OF WORDS

Groups of organising concepts and associated database search terms

A. Conscientious Objection	B. Euthanasia and Assisted Suicide
Conscientious; conscience; religious object*; moral object*; ethical object*; religious conflict*; moral conflict*; ethical conflict*; refusal*; exemption*; dilemma*; patient abandonment; contestant*; dissenter*; dissident*; objector*; protester*; non participation; non compliance	euthan*; mercy killing*; assisted suicide*; assisted dying; assistance in dying; assisted death*; death with dignity; dying with dignity; right to die; aid in dying; MAID; hastening death*; hastened death*; end of life decision*; end of life choice*; end of life right*; end of life option act*; life-ending intervention*; compassionate death*; life ending act*; life ending decision*; active life termination*; termination of life on request*

DATABASES

Number of positive results (“hits”) in each database. Search date 19/09/2024.

Field	Database		Date	Results
Biomedical	1.	Pubmed	19/09/2024	1974
	2.	Embase	19/09/2024	1618
	3.	Web of Science	19/09/2024	973
	4.	CINAHL	19/09/2024	781
Multidisciplinary Sciences	5.	SciELO	19/09/2024	57
	6.	Scopus	19/09/2024	1394
	7.	ProQuest Central	19/09/2024	747
Philosophy	8.	The Philosopher’s Index	19/09/2024	220
	9.	JSTOR	19/09/2024	605
	10.	PhilPapers	19/09/2024	375
Theology	11.	Atla	19/09/2024	42
	12.	Index Religiosus	19/09/2024	10
	13.	Index Theologicus	19/09/2024	208
			Total	9004

PUBMED (via NCBI, including MEDLINE)

In PubMed Advanced Search Builder

Group A

"Refusal to Treat"[Mesh] OR "Conscience"[Mesh] OR "Refusal to Participate"[Mesh] OR “conscientious”[tiab] OR “conscience”[tiab] OR “religious object*”[tiab] OR “moral object*”[tiab] OR “ethical object*”[tiab] OR “religious conflict*”[tiab] OR “moral conflict*”[tiab] OR “ethical conflict*”[tiab] OR “refusal*”[tiab] OR “exemption*”[tiab] OR “dilemma*”[tiab] OR “patient abandonment”[tiab] OR “contestant*”[tiab] OR “dissenter*”[tiab] OR “dissident*”[tiab] OR “objector*”[tiab] OR “protester*”[tiab] OR “non participation”[tiab] OR “non compliance”[tiab]

Hits: 86,914

Group B

"Euthanasia"[Mesh] OR "Right to Die"[Mesh] OR "Suicide, Assisted"[Mesh] OR “euthan*”[tiab] OR “mercy killing*”[tiab] OR “assisted suicide*”[tiab] OR “assisted dying”[tiab] OR “assistance in dying”[tiab] OR “assisted death*”[tiab] OR “death with dignity”[tiab] OR “dying with dignity”[tiab] OR “right to die”[tiab] OR “aid in dying”[tiab] OR “MAID”[tiab] OR “hastening death*”[tiab] OR “hastened death*”[tiab] OR “end of life decision*”[tiab] OR “end of life choice*”[tiab] OR “end of life right*”[tiab] OR “end of life option act*”[tiab] OR “life-ending intervention*”[tiab] OR “compassionate death*”[tiab] OR “life ending act*”[tiab] OR “life ending decision*”[tiab] OR “active life termination*”[tiab] OR “termination of life on request*”[tiab]

Hits: 61,186

Combined Search: A AND B

("Refusal to Treat"[Mesh] OR "Conscience"[Mesh] OR "Refusal to Participate"[Mesh] OR “conscientious”[tiab] OR “conscience”[tiab] OR “religious object*”[tiab] OR “moral object*”[tiab] OR “ethical object*”[tiab] OR “religious conflict*”[tiab] OR “moral conflict*”[tiab] OR “ethical conflict*”[tiab] OR “refusal*”[tiab] OR “exemption*”[tiab] OR “dilemma*”[tiab] OR “patient abandonment”[tiab] OR “contestant*”[tiab] OR “dissenter*”[tiab] OR “dissident*”[tiab] OR “objector*”[tiab] OR “protester*”[tiab] OR “non participation”[tiab] OR “non compliance”[tiab]) AND ("Euthanasia"[Mesh] OR "Right to Die"[Mesh] OR "Suicide, Assisted"[Mesh] OR “euthan*”[tiab] OR “mercy killing*”[tiab] OR “assisted suicide*”[tiab] OR “assisted dying”[tiab] OR “assistance in dying”[tiab] OR “assisted death*”[tiab] OR “death with dignity”[tiab] OR “dying with dignity”[tiab] OR “right to die”[tiab] OR “aid in dying”[tiab] OR “MAID”[tiab] OR “hastening death*”[tiab] OR “hastened death*”[tiab] OR “end of life decision*”[tiab] OR “end of life choice*”[tiab] OR “end of life right*”[tiab] OR “end of life option act*”[tiab] OR “life-ending intervention*”[tiab] OR “compassionate death*”[tiab] OR “life ending act*”[tiab] OR “life ending decision*”[tiab] OR “active life termination*”[tiab] OR “termination of life on request*”[tiab])

Results: 1,974

EMBASE (via Embase.com)

In Advanced Search. Unchecked: “Embase mapping options”.

Group A

'conscientious objection'/exp OR 'refusal to participate'/exp OR 'patient abandonment'/exp OR 'conscience'/exp OR 'ethical dilemma'/exp OR 'conscientious':ti,ab,kw OR 'conscience':ti,ab,kw OR 'religious object*':ti,ab,kw OR 'moral object*':ti,ab,kw OR 'ethical object*':ti,ab,kw OR 'religious conflict*':ti,ab,kw OR 'moral conflict*':ti,ab,kw OR 'ethical conflict*':ti,ab,kw OR 'refusal*':ti,ab, kw OR 'exemption*':ti,ab,kw OR 'dilemma*':ti,ab,kw OR 'patient abandonment':ti,ab,kw OR 'contestant*':ti,ab,kw OR 'dissenter*':ti,ab,kw OR 'dissident*':ti,ab,kw OR 'objector*':ti,ab,kw OR 'protester*':ti,ab,kw OR 'non participation':ti,ab,kw OR 'non compliance':ti,ab,kw

Hits: 120.554

Group B

'euthanasia'/exp OR 'assisted suicide'/exp OR 'right to die'/exp OR 'end of life decision'/exp OR ‘euthan*’:ti,ab,kw OR ‘mercy killing*’:ti,ab,kw OR ‘assisted suicide*’:ti,ab,kw OR ‘assisted dying’:ti,ab,kw OR ‘assistance in dying’:ti,ab,kw OR ‘assisted death*’:ti,ab,kw OR ‘death with dignity’:ti,ab,kw OR ‘dying with dignity’:ti,ab,kw OR ‘right to die’:ti,ab,kw OR ‘aid in dying’:ti,ab,kw OR ‘MAID’:ti,ab,kw OR ‘hastening death*’:ti,ab,kw OR ‘hastened death*’:ti,ab,kw OR ‘end of life decision*’:ti,ab,kw OR ‘end of life choice*’:ti,ab,kw OR ‘end of life right*’:ti,ab,kw OR ‘end of life option act*’:ti,ab,kw OR ‘life-ending intervention*’:ti,ab,kw OR ‘compassionate death*’:ti,ab,kw OR ‘life ending act*’:ti,ab,kw OR ‘life ending decision*’:ti,ab,kw OR ‘active life termination*’:ti,ab,kw OR ‘termination of life on request*’:ti,ab,kw

Hits: 78.946

Combined Search: A AND B

('conscientious objection'/exp OR 'refusal to participate'/exp OR 'patient abandonment'/exp OR 'conscience'/exp OR 'ethical dilemma'/exp OR 'conscientious':ti,ab,kw OR 'conscience':ti,ab,kw OR 'religious object*':ti,ab,kw OR 'moral object*':ti,ab,kw OR 'ethical object*':ti,ab,kw OR 'religious conflict*':ti,ab,kw OR 'moral conflict*':ti,ab,kw OR 'ethical conflict*':ti,ab,kw OR 'refusal*':ti,ab,kw OR 'exemption*':ti,ab,kw OR 'dilemma*':ti,ab,kw OR 'patient abandonment':ti,ab,kw OR 'contestant*':ti,ab,kw OR 'dissenter*':ti,ab,kw OR 'dissident*':ti,ab,kw OR 'objector*':ti,ab,kw OR 'protester*':ti,ab,kw OR 'non participation':ti,ab,kw OR 'non compliance':ti,ab,kw) AND ('euthanasia'/exp OR 'assisted suicide'/exp OR 'right to die'/exp OR 'end of life decision'/exp OR ‘euthan*’:ti,ab,kw OR ‘mercy killing*’:ti,ab,kw OR ‘assisted suicide*’:ti,ab,kw OR ‘assisted dying’:ti,ab,kw OR ‘assistance in dying’:ti,ab,kw OR ‘assisted death*’:ti,ab,kw OR ‘death with dignity’:ti,ab,kw OR ‘dying with dignity’:ti,ab,kw OR ‘right to die’:ti,ab,kw OR ‘aid in dying’:ti,ab,kw OR ‘MAID’:ti,ab,kw OR ‘hastening death*’:ti,ab,kw OR ‘hastened death*’:ti,ab,kw OR ‘end of life decision*’:ti,ab,kw OR ‘end of life choice*’:ti,ab,kw OR ‘end of life right*’:ti,ab,kw OR ‘end of life option act*’:ti,ab,kw OR ‘life-ending intervention*’:ti,ab,kw OR ‘compassionate death*’:ti,ab,kw OR ‘life ending act*’:ti,ab,kw OR ‘life ending decision*’:ti,ab,kw OR ‘active life termination*’:ti,ab,kw OR ‘termination of life on request*’:ti,ab,kw) NOT 'conference abstract':it

Results: 1.618

WEB OF SCIENCE (WoS Core Collection; via webofscience.com)

In Advanced Search. Indexes used:

- *Science Citation Index Expanded (SCI-EXPANDED)--1955-present*
- *Social Sciences Citation Index (SSCI)--1956-present*
- *Arts & Humanities Citation Index (AHCI)--1975-present*
- *Conference Proceedings Citation Index – Science (CPCI-S)--1990-present*
- *Conference Proceedings Citation Index – Social Science & Humanities (CPCI-SSH)--1990-present*
- *Emerging Sources Citation Index (ESCI)--2019-present*

Group A

TS=("conscientious" OR "conscience" OR "religious object*" OR "moral object*" OR "ethical object*" OR "religious conflict*" OR "moral conflict*" OR "ethical conflict*" OR "refusal*" OR "exemption*" OR "dilemma*" OR "patient abandonment" OR "contestant*" OR "dissenter*" OR "dissident*" OR "objector*" OR "protester*" OR "non participation" OR "non compliance")

Hits: 167.165

Group B:

TS=("euthan*" OR "mercy killing*" OR "assisted suicide*" OR "assisted dying" OR "assistance in dying" OR "assisted death*" OR "death with dignity" OR "dying with dignity" OR "right to die" OR "aid in dying" OR "MAID" OR "hastening death*" OR "hastened death*" OR "end of life decision*" OR "end of life choice*" OR "end of life right*" OR "end of life option act*" OR "life-ending intervention*" OR "compassionate death*" OR "life ending act*" OR "life ending decision*" OR "active life termination*" OR "termination of life on request*")

Hits: 49.142

Combined Search: A AND B

TS=("conscientious" OR "conscience" OR "religious object*" OR "moral object*" OR "ethical object*" OR "religious conflict*" OR "moral conflict*" OR "ethical conflict*" OR "refusal*" OR "exemption*" OR "dilemma*" OR "patient abandonment" OR "contestant*" OR "dissenter*" OR "dissident*" OR "objector*" OR "protester*" OR "non participation" OR "non compliance") AND TS=("euthan*" OR "mercy killing*" OR "assisted suicide*" OR "assisted dying" OR "assistance in dying" OR "assisted death*" OR "death with dignity" OR "dying with dignity" OR "right to die" OR "aid in dying" OR "MAID" OR "hastening death*" OR "hastened death*" OR "end of life decision*" OR "end of life choice*" OR "end of life right*" OR "end of life option act*" OR "life-ending intervention*" OR "compassionate death*" OR "life ending act*" OR "life ending decision*" OR "active life termination*" OR "termination of life on request*")

Results: 973

CINAHL (via EBSCOhost)

In Advanced Search.

Group A

(MH "Refusal to Treat") OR (MH "Refusal to Participate") OR (MH "Conscience") OR (MH "Patient Abandonment") OR TI (“conscientious” OR “conscience” OR “religious object*” OR “moral object*” OR “ethical object*” OR “religious conflict*” OR “moral conflict*” OR “ethical conflict*” OR “refusal*” OR “exemption*” OR “dilemma*” OR “patient abandonment” OR “contestant*” OR “dissenter*” OR “dissident*” OR “objector*” OR “protester*” OR “non participation” OR “non compliance”) OR AB (“conscientious” OR “conscience” OR “religious object*” OR “moral object*” OR “ethical object*” OR “religious conflict*” OR “moral conflict*” OR “ethical conflict*” OR “refusal*” OR “exemption*” OR “dilemma*” OR “patient abandonment” OR “contestant*” OR “dissenter*” OR “dissident*” OR “objector*” OR “protester*” OR “non participation” OR “non compliance”)

Hits: 32,159

Group B:

(MH "Euthanasia+") OR (MH "Right to Die") OR (MH "Suicide, Assisted") OR TI (“euthan*” OR “mercy killing*” OR “assisted suicide*” OR “assisted dying” OR “assistance in dying” OR “assisted death*” OR “death with dignity” OR “dying with dignity” OR “right to die” OR “aid in dying” OR “MAID” OR “hastening death*” OR “hastened death*” OR “end of life decision*” OR “end of life choice*” OR “end of life right*” OR “end of life option act*” OR “life-ending intervention*” OR “compassionate death*” OR “life ending act*” OR “life ending decision*” OR “active life termination*” OR “termination of life on request*”) OR AB (“euthan*” OR “mercy killing*” OR “assisted suicide*” OR “assisted dying” OR “assistance in dying” OR “assisted death*” OR “death with dignity” OR “dying with dignity” OR “right to die” OR “aid in dying” OR “MAID” OR “hastening death*” OR “hastened death*” OR “end of life decision*” OR “end of life choice*” OR “end of life right*” OR “end of life option act*” OR “life-ending intervention*” OR “compassionate death*” OR “life ending act*” OR “life ending decision*” OR “active life termination*” OR “termination of life on request*”)

Hits: 17,295

Combined Search: A AND B

((MH "Refusal to Treat") OR (MH "Refusal to Participate") OR (MH "Conscience") OR (MH "Patient Abandonment") OR TI (“conscientious” OR “conscience” OR “religious object*” OR “moral object*” OR “ethical object*” OR “religious conflict*” OR “moral conflict*” OR “ethical conflict*” OR “refusal*” OR “exemption*” OR “dilemma*” OR “patient abandonment” OR “contestant*” OR “dissenter*” OR “dissident*” OR “objector*” OR “protester*” OR “non participation” OR “non compliance”) OR AB (“conscientious” OR “conscience” OR “religious object*” OR “moral object*” OR “ethical object*” OR “religious conflict*” OR “moral conflict*” OR “ethical conflict*” OR “refusal*” OR “exemption*” OR “dilemma*” OR “patient abandonment” OR “contestant*” OR “dissenter*” OR “dissident*” OR “objector*” OR “protester*” OR “non participation” OR “non compliance”)) AND ((MH "Euthanasia+") OR (MH "Right to Die") OR (MH "Suicide, Assisted") OR TI (“euthan*” OR “mercy killing*” OR “assisted suicide*” OR “assisted dying” OR “assistance in dying” OR “assisted death*” OR “death with dignity” OR “dying with dignity” OR “right to die” OR “aid in dying” OR “MAID” OR “hastening death*” OR “hastened death*” OR “end of life decision*” OR “end of life choice*” OR “end of life right*” OR “end of life option act*” OR “life-ending intervention*” OR “compassionate death*” OR “life ending act*” OR “life ending decision*” OR “active life termination*” OR “termination of life on request*”) OR AB (“euthan*” OR “mercy killing*” OR “assisted suicide*” OR “assisted dying” OR “assistance in dying” OR “assisted death*” OR “death with dignity” OR “dying with dignity” OR “right to die” OR “aid in dying” OR “MAID” OR “hastening death*” OR “hastened death*” OR “end of life decision*” OR “end of life choice*” OR “end of life right*” OR “end of life option act*” OR “life-ending intervention*” OR “compassionate death*” OR “life ending act*” OR “life ending decision*” OR “active life termination*” OR “termination of life on request*”))

Results: 781

SCIELO (via webofscience.com)

In Advanced Search Query Builder:

Group A

TS=("conscientious" OR "conscience" OR "religious object*" OR "moral object*" OR "ethical object*" OR "religious conflict*" OR "moral conflict*" OR "ethical conflict*" OR "refusal*" OR "exemption*" OR "dilemma*" OR "patient abandonment" OR "contestant*" OR "dissenter*" OR "dissident*" OR "objector*" OR "protester*" OR "non participation" OR "non compliance")

Hits: 6,748

Group B:

TS=("euthan*" OR "mercy killing*" OR "assisted suicide*" OR "assisted dying" OR "assistance in dying" OR "assisted death*" OR "death with dignity" OR "dying with dignity" OR "right to die" OR "aid in dying" OR "MAID" OR "hastening death*" OR "hastened death*" OR "end of life decision*" OR "end of life choice*" OR "end of life right*" OR "end of life option act*" OR "life-ending intervention*" OR "compassionate death*" OR "life ending act*" OR "life ending decision*" OR "active life termination*" OR "termination of life on request*")

Hits: 2,026

Combined Search: A AND B

TS=("conscientious" OR "conscience" OR "religious object*" OR "moral object*" OR "ethical object*" OR "religious conflict*" OR "moral conflict*" OR "ethical conflict*" OR "refusal*" OR "exemption*" OR "dilemma*" OR "patient abandonment" OR "contestant*" OR "dissenter*" OR "dissident*" OR "objector*" OR "protester*" OR "non participation" OR "non compliance") AND TS=("euthan*" OR "mercy killing*" OR "assisted suicide*" OR "assisted dying" OR "assistance in dying" OR "assisted death*" OR "death with dignity" OR "dying with dignity" OR "right to die" OR "aid in dying" OR "MAID" OR "hastening death*" OR "hastened death*" OR "end of life decision*" OR "end of life choice*" OR "end of life right*" OR "end of life option act*" OR "life-ending intervention*" OR "compassionate death*" OR "life ending act*" OR "life ending decision*" OR "active life termination*" OR "termination of life on request*")

Results: 57

SCOPUS (via scopus.com)

In Advanced Document Search.

Group A

TITLE-ABS ("conscientious" OR "conscience" OR "religious object*" OR "moral object*" OR "ethical object*" OR "religious conflict*" OR "moral conflict*" OR "ethical conflict*" OR "refusal*" OR "exemption*" OR "dilemma*" OR "patient abandonment" OR "contestant*" OR "dissenter*" OR "dissident*" OR "objector*" OR "protester*" OR "non participation" OR "non compliance") OR AUTHKEY ("conscientious" OR "conscience" OR "religious object*" OR "moral object*" OR "ethical object*" OR "religious conflict*" OR "moral conflict*" OR "ethical conflict*" OR "refusal*" OR "exemption*" OR "dilemma*" OR "patient abandonment" OR "contestant*" OR "dissenter*" OR "dissident*" OR "objector*" OR "protester*" OR "non participation" OR "non compliance")

Hits: 232,585

Group B:

TITLE-ABS ("euthan*" OR "mercy killing*" OR "assisted suicide*" OR "assisted dying" OR "assistance in dying" OR "assisted death*" OR "death with dignity" OR "dying with dignity" OR "right to die" OR "aid in dying" OR "MAID" OR "hastening death*" OR "hastened death*" OR "end of life decision*" OR "end of life choice*" OR "end of life right*" OR "end of life option act*" OR "life-ending intervention*" OR "compassionate death*" OR "life ending act*" OR "life ending decision*" OR "active life termination*" OR "termination of life on request*") OR AUTHKEY ("euthan*" OR "mercy killing*" OR "assisted suicide*" OR "assisted dying" OR "assistance in dying" OR "assisted death*" OR "death with dignity" OR "dying with dignity" OR "right to die" OR "aid in dying" OR "MAID" OR "hastening death*" OR "hastened death*" OR "end of life decision*" OR "end of life choice*" OR "end of life right*" OR "end of life option act*" OR "life-ending intervention*" OR "compassionate death*" OR "life ending act*" OR "life ending decision*" OR "active life termination*" OR "termination of life on request*")

Hits: 59,485

Combined Search: A AND B

(TITLE-ABS ("conscientious" OR "conscience" OR "religious object*" OR "moral object*" OR "ethical object*" OR "religious conflict*" OR "moral conflict*" OR "ethical conflict*" OR "refusal*" OR "exemption*" OR "dilemma*" OR "patient abandonment" OR "contestant*" OR "dissenter*" OR "dissident*" OR "objector*" OR "protester*" OR "non participation" OR "non compliance") OR AUTHKEY ("conscientious" OR "conscience" OR "religious object*" OR "moral object*" OR "ethical object*" OR "religious conflict*" OR "moral conflict*" OR "ethical conflict*" OR "refusal*" OR "exemption*" OR "dilemma*" OR "patient abandonment" OR "contestant*" OR "dissenter*" OR "dissident*" OR "objector*" OR "protester*" OR "non participation" OR "non compliance")) AND (TITLE-ABS ("euthan*" OR "mercy killing*" OR "assisted suicide*" OR "assisted dying" OR "assistance in dying" OR "assisted death*" OR "death with dignity" OR "dying with dignity" OR "right to die" OR "aid in dying" OR "MAID" OR "hastening death*" OR "hastened death*" OR "end of life decision*" OR "end of life choice*" OR "end of life right*" OR "end of life option act*" OR "life-ending intervention*" OR "compassionate death*" OR "life ending act*" OR "life ending decision*" OR "active life termination*" OR "termination of life on request*") OR AUTHKEY ("euthan*" OR "mercy killing*" OR "assisted suicide*" OR "assisted dying" OR "assistance in dying" OR "assisted death*" OR "death with dignity" OR "dying with dignity" OR "right to die" OR "aid in dying" OR "MAID" OR "hastening death*" OR "hastened death*" OR "end of life decision*" OR "end of life choice*" OR "end of life right*" OR "end of life option act*" OR "life-ending intervention*" OR "compassionate death*" OR "life ending act*" OR "life ending decision*" OR "active life termination*" OR "termination of life on request*"))

Results: 1,394

PROQUEST CENTRAL (via ProQuest)

In Advanced Search.

Group A

MAINSUBJECT.EXACT("Conscientious objectors" OR "Religious exemptions") OR TI,AB,IF(“conscientious” OR “conscience” OR “religious object*” OR “moral object*” OR “ethical object*” OR “religious conflict*” OR “moral conflict*” OR “ethical conflict*” OR “refusal*” OR “exemption*” OR “dilemma*” OR “patient abandonment” OR “contestant*” OR “dissenter*” OR “dissident*” OR “objector*” OR “protester*” OR “non participation” OR “non compliance”)

Hits: 103.606

Group B

MAINSUBJECT.EXACT("Euthanasia" OR “Assisted suicide”) OR TI,AB,IF(“euthan*” OR “mercy killing*” OR “assisted suicide*” OR “assisted dying” OR “assistance in dying” OR “assisted death*” OR “death with dignity” OR “dying with dignity” OR “right to die” OR “aid in dying” OR “MAID” OR “hastening death*” OR “hastened death*” OR “end of life decision*” OR “end of life choice*” OR “end of life right*” OR “end of life option act*” OR “life-ending intervention*” OR “compassionate death*” OR “life ending act*” OR “life ending decision*” OR “active life termination*” OR “termination of life on request*”)

Hits: 23.561

Combined Search: A AND B

(MAINSUBJECT.EXACT("Conscientious objectors" OR "Religious exemptions") OR TI,AB,IF(“conscientious” OR “conscience” OR “religious object*” OR “moral object*” OR “ethical object*” OR “religious conflict*” OR “moral conflict*” OR “ethical conflict*” OR “refusal*” OR “exemption*” OR “dilemma*” OR “patient abandonment” OR “contestant*” OR “dissenter*” OR “dissident*” OR “objector*” OR “protester*” OR “non participation” OR “non compliance”)) AND (MAINSUBJECT.EXACT("Euthanasia" OR “Assisted suicide”) OR TI,AB,IF(“euthan*” OR “mercy killing*” OR “assisted suicide*” OR “assisted dying” OR “assistance in dying” OR “assisted death*” OR “death with dignity” OR “dying with dignity” OR “right to die” OR “aid in dying” OR “MAID” OR “hastening death*” OR “hastened death*” OR “end of life decision*” OR “end of life choice*” OR “end of life right*” OR “end of life option act*” OR “life-ending intervention*” OR “compassionate death*” OR “life ending act*” OR “life ending decision*” OR “active life termination*” OR “termination of life on request*”))

Results:747

THE PHILOSOPHER'S INDEX (via OVID)

Advanced Search. Unchecked: "include related terms"

Group A

("conscientious" OR "conscience" OR "religious object*" OR "moral object*" OR "ethical object*" OR "religious conflict*" OR "moral conflict*" OR "ethical conflict*" OR "refusal*" OR "exemption*" OR "dilemma*" OR "patient abandonment" OR "contestant*" OR "dissenter*" OR "dissident*" OR "objector*" OR "protester*" OR "non participation" OR "non compliance").ti,ab,hw.

Hits: 13705

Group B

("euthan*" OR "mercy killing*" OR "assisted suicide*" OR "assisted dying" OR "assistance in dying" OR "assisted death*" OR "death with dignity" OR "dying with dignity" OR "right to die" OR "aid in dying" OR "MAID" OR "hastening death*" OR "hastened death*" OR "end of life decision*" OR "end of life choice*" OR "end of life right*" OR "end of life option act*" OR "life-ending intervention*" OR "compassionate death*" OR "life ending act*" OR "life ending decision*" OR "active life termination*" OR "termination of life on request*").ti,ab,hw.

Hits: 2631

Combined Search: A AND B

("conscientious" OR "conscience" OR "religious object*" OR "moral object*" OR "ethical object*" OR "religious conflict*" OR "moral conflict*" OR "ethical conflict*" OR "refusal*" OR "exemption*" OR "dilemma*" OR "patient abandonment" OR "contestant*" OR "dissenter*" OR "dissident*" OR "objector*" OR "protester*" OR "non participation" OR "non compliance").ti,ab,hw. AND ("euthan*" OR "mercy killing*" OR "assisted suicide*" OR "assisted dying" OR "assistance in dying" OR "assisted death*" OR "death with dignity" OR "dying with dignity" OR "right to die" OR "aid in dying" OR "MAID" OR "hastening death*" OR "hastened death*" OR "end of life decision*" OR "end of life choice*" OR "end of life right*" OR "end of life option act*" OR "life-ending intervention*" OR "compassionate death*" OR "life ending act*" OR "life ending decision*" OR "active life termination*" OR "termination of life on request*").ti,ab,hw.

Results: 220

JSTOR (via JSTOR.org)

**Only allows a limited boolean search*.

Filters used:

- *Access type: "Everything"*
- *Academic content: "Journals"*

Group A

("conscientious objection" OR "patient abandonment" OR "refusal to treat" OR objector*)

Group B

(euthan* OR "assisted suicide" OR "mercy killing" OR "death with dignity" OR "right to die" OR "hastening death" OR "end of life decision")

Combined Search: A AND B

("conscientious objection" OR "patient abandonment" OR "refusal to treat" OR objector*) AND (euthan* OR "assisted suicide" OR "mercy killing" OR "death with dignity" OR "right to die" OR "hastening death" OR "end of life decision")

Results: 605

PHIL PAPERS (via philpapers.org)

**Only allows a limited boolean search*.

In Advanced search, search string entered in “matching this extended query”:

Group A

("conscien*"|refus*| conflict*| objection*|"patient abandonment")

Group B

(euthan*|"assisted suicide" | "right to die")

Combined Search: A AND B

("conscien*"|refus*| conflict*| objection*|"patient abandonment")&(euthan*|"assisted suicide" | "right to die")

Results: 375

ATLA RELIGION DATABASE WITH ATLASERIALS PLUS (via EBSCOhost)

In Advanced Search

Group A

DE ("Conscientious objection" OR "Conscientious objectors" "Refusal to treat") OR TI (“conscientious” OR “conscience” OR “religious object*” OR “moral object*” OR “ethical object*” OR “religious conflict*” OR “moral conflict*” OR “ethical conflict*” OR “refusal*” OR “exemption*” OR “dilemma*” OR “patient abandonment” OR “contestant*” OR “dissenter*” OR “dissident*” OR “objector*” OR “protester*” OR “non participation” OR “non compliance”) OR AB (“conscientious” OR “conscience” OR “religious object*” OR “moral object*” OR “ethical object*” OR “religious conflict*” OR “moral conflict*” OR “ethical conflict*” OR “refusal*” OR “exemption*” OR “dilemma*” OR “patient abandonment” OR “contestant*” OR “dissenter*” OR “dissident*” OR “objector*” OR “protester*” OR “non participation” OR “non compliance”)

Hits: 10,563

Group B

DE ("Euthanasia" OR "Assisted suicide" OR "Right to die") OR TI (“euthan*” OR “mercy killing*” OR “assisted suicide*” OR “assisted dying” OR “assistance in dying” OR “assisted death*” OR “death with dignity” OR “dying with dignity” OR “right to die” OR “aid in dying” OR “MAID” OR “hastening death*” OR “hastened death*” OR “end of life decision*” OR “end of life choice*” OR “end of life right*” OR “end of life option act*” OR “life-ending intervention*” OR “compassionate death*” OR “life ending act*” OR “life ending decision*” OR “active life termination*” OR “termination of life on request*”) OR AB (“euthan*” OR “mercy killing*” OR “assisted suicide*” OR “assisted dying” OR “assistance in dying” OR “assisted death*” OR “death with dignity” OR “dying with dignity” OR “right to die” OR “aid in dying” OR “MAID” OR “hastening death*” OR “hastened death*” OR “end of life decision*” OR “end of life choice*” OR “end of life right*” OR “end of life option act*” OR “life-ending intervention*” OR “compassionate death*” OR “life ending act*” OR “life ending decision*” OR “active life termination*” OR “termination of life on request*”)

Hits: 3,013

Combined Search: A AND B

(DE ("Conscientious objection" OR "Conscientious objectors" "Refusal to treat") OR TI (“conscientious” OR “conscience” OR “religious object*” OR “moral object*” OR “ethical object*” OR “religious conflict*” OR “moral conflict*” OR “ethical conflict*” OR “refusal*” OR “exemption*” OR “dilemma*” OR “patient abandonment” OR “contestant*” OR “dissenter*” OR “dissident*” OR “objector*” OR “protester*” OR “non participation” OR “non compliance”) OR AB (“conscientious” OR “conscience” OR “religious object*” OR “moral object*” OR “ethical object*” OR “religious conflict*” OR “moral conflict*” OR “ethical conflict*” OR “refusal*” OR “exemption*” OR “dilemma*” OR “patient abandonment” OR “contestant*” OR “dissenter*” OR “dissident*” OR “objector*” OR “protester*” OR “non participation” OR “non compliance”)) AND (DE ("Euthanasia" OR "Assisted suicide" OR "Right to die") OR TI (“euthan*” OR “mercy killing*” OR “assisted suicide*” OR “assisted dying” OR “assistance in dying” OR “assisted death*” OR “death with dignity” OR “dying with dignity” OR “right to die” OR “aid in dying” OR “MAID” OR “hastening death*” OR “hastened death*” OR “end of life decision*” OR “end of life choice*” OR “end of life right*” OR “end of life option act*” OR “life-ending intervention*” OR “compassionate death*” OR “life ending act*” OR “life ending decision*” OR “active life termination*” OR “termination of life on request*”) OR AB (“euthan*” OR “mercy killing*” OR “assisted suicide*” OR “assisted dying” OR “assistance in dying” OR “assisted death*” OR “death with dignity” OR “dying with dignity” OR “right to die” OR “aid in dying” OR “MAID” OR “hastening death*” OR “hastened death*” OR “end of life decision*” OR “end of life choice*” OR “end of life right*” OR “end of life option act*” OR “life-ending intervention*” OR “compassionate death*” OR “life ending act*” OR “life ending decision*” OR “active life termination*” OR “termination of life on request*”))

Results: 42

INDEX RELIGIOUS (via BREPOLiS)

In Simple Search.

Group A

(“conscientious” OR “conscience” OR “religious object*” OR “moral object*” OR “ethical object*” OR “religious conflict*” OR “moral conflict*” OR “ethical conflict*” OR “refusal*” OR “exemption*” OR “dilemma*” OR “patient abandonment” OR “contestant*” OR “dissenter*” OR “dissident*” OR “objector*” OR “protester*” OR “non participation” OR “non compliance”)

Hits: 3084

Group B

(“euthan*” OR “mercy killing*” OR “assisted suicide*” OR “assisted dying” OR “assistance in dying” OR “assisted death*” OR “death with dignity” OR “dying with dignity” OR “right to die” OR “aid in dying” OR “MAID” OR “hastening death*” OR “hastened death*” OR “end of life decision*” OR “end of life choice*” OR “end of life right*” OR “end of life option act*” OR “life-ending intervention*” OR “compassionate death*” OR “life ending act*” OR “life ending decision*” OR “active life termination*” OR “termination of life on request*”)

Hits: 595

Combined Search: A AND B

(“conscientious” OR “conscience” OR “religious object*” OR “moral object*” OR “ethical object*” OR “religious conflict*” OR “moral conflict*” OR “ethical conflict*” OR “refusal*” OR “exemption*” OR “dilemma*” OR “patient abandonment” OR “contestant*” OR “dissenter*” OR “dissident*” OR “objector*” OR “protester*” OR “non participation” OR “non compliance”) AND (“euthan*” OR “mercy killing*” OR “assisted suicide*” OR “assisted dying” OR “assistance in dying” OR “assisted death*” OR “death with dignity” OR “dying with dignity” OR “right to die” OR “aid in dying” OR “MAID” OR “hastening death*” OR “hastened death*” OR “end of life decision*” OR “end of life choice*” OR “end of life right*” OR “end of life option act*” OR “life-ending intervention*” OR “compassionate death*” OR “life ending act*” OR “life ending decision*” OR “active life termination*” OR “termination of life on request*”)

Results: 10

INDEX THEOLOGICUS (via ixtheo.de)

In Advanced Search.

Group A

(conscientious OR conscience OR objector* OR objection* OR “religious conflict” OR “moral conflict” OR “ethical conflict” OR refusal* OR exemption* OR dilemma* OR “patient abandonment” OR contestant* OR dissenter* OR dissident* OR protester* OR “non participation” OR “non compliance”)

Hits: 25,252

Group B

(euthan* OR “mercy killing” OR “assisted suicide” OR “assisted dying” OR “assistance in dying” OR “assisted death” OR “death with dignity” OR “dying with dignity” OR “right to die” OR “aid in dying” OR “MAID” OR “hastening death” OR “hastened death” OR “end of life decision” OR “end of life choice” OR “end of life right” OR “end of life option act” OR “life-ending intervention” OR “compassionate death” OR “life ending act” OR “life ending decision” OR “active life termination” OR “termination of life on request”)

Hits: 4,148

Combined Search: A AND B

(conscientious OR conscience OR objector* OR objection* OR “religious conflict” OR “moral conflict” OR “ethical conflict” OR refusal* OR exemption* OR dilemma* OR “patient abandonment” OR contestant* OR dissenter* OR dissident* OR protester* OR “non participation” OR “non compliance”) AND (euthan* OR “mercy killing” OR “assisted suicide” OR “assisted dying” OR “assistance in dying” OR “assisted death” OR “death with dignity” OR “dying with dignity” OR “right to die” OR “aid in dying” OR “MAID” OR “hastening death” OR “hastened death” OR “end of life decision” OR “end of life choice” OR “end of life right” OR “end of life option act” OR “life-ending intervention” OR “compassionate death” OR “life ending act” OR “life ending decision” OR “active life termination” OR “termination of life on request”)

Results: 208
