# Supplementary material for: Conscientious objection in euthanasia and assisted suicide: A systematic review
Source: PLoS One. 2025 Jun 23;20(6):e0326142. doi: 10.1371/journal.pone.0326142 (PMC12185019; doi:10.1371/journal.pone.0326142)
Supplement: S6 File — (PDF) [file pone.0326142.s006.pdf]

**X**	Brassfield, E. R., Mishra, M., & Buchbinder, M. (2019). Responding to requests for aid-in-dying: Rethinking the role of conscience. *Narrative Inquiry in Bioethics*, *9*(1), 67-72.
**Topic & Comment**	A deep article that, through a case study, begins by analysing traditional views on conscience and proposes a more nuanced and dynamic perspective. It advocates for the development of certain skills to help healthcare professionals navigate conscience conflicts
**Country**	USA<br>Philosophy and Medicine
**CO Taxonomy**	☐ Absolutism CO X Compromise ☐ Incompatibility ☐ Not applicable
**EAS Taxonomy**	☐ Full reject X Conditioned S/R ☐ Full Support ☐ Not applicable
**Objector**	X Physician ☐ Nurse ☐ Pharmacist ☐ HCW in general<br>☐ Institutions ☐ Others:
**Addressed topics**	X Referral X Information X Disclosure ☐ Register<br>X Moral integrity X Moral complicity ☐ Criteria for acceptability
**Ethical Approach**	Care ethics. Principlism. Relational ethics. Ethics of responsibility.
**Definition of CO**	conscience clauses protect providers' rights to refuse to participate in medical services for moral or religious reasons (p. 70)
**Focus on EAS**	"Aid-in-dying" (AID) statutes permitting a physician to write a prescription for a lethal dose of medication to a terminally ill adult patient for the purpose of ending that patient's life
**Fundamental concepts**	Conscience.
**Religious arguments**	CO is not always based on an unwavering moral conviction. Most physicians lack a perspicuous ordering or weighting system for the values they hold.
**Supporting Authors**	Long list of supporting authors.
**Bibliography track**	Brock, D. (2008); Giubilini, A. (2014);

Classical approach to Conscience:

- A reflection on how actions align or conflict with core moral principles and values; along with a commitment to act in accordance with these moral judgements. (Childress 1979)
- Informed by religious or secular beliefs (Lawrence and Curlin, 2007)
- Essential to the ethical practice of medicine (Sulmasy 2008)
- Characterization: absolutism CO; incompatibility thesis; conventional compromise (Wicclair 2011)
- Conflicts of conscience are largely conceptualized in terms of threats to moral integrity (Wicclair 2000)

An **alternative** approach:

- Dynamism: an evolving product shaped by ongoing moral judgements (Childress, Charo, Guibulini).
- Relational: conscience encourages morally responsible agency attuned to the welfare of others (Mc Leod)
  - Forcing us to rethink our values or stretch our horizons regarding ethically acceptable behaviour
- Open space for more positive formulations of conscience that emphasize commitment to care and responsibility for others (Belinguer 2016)

A simplistic view of CO can be easily resolved by implementing policies that protect physicians' rights to object.

- Sometimes the law may condone practices that the clinicians object
- Other times, clinicians may feel compelled to provide services that the law forbids
- Often, clinicians find themselves divided, holding values that come into conflict

Case study: *a complex and nuanced real-life situation about a physician who receives a PAD request coming a long-term patient with whom they share a significant history.*

ETHICAL ANALYSIS of the case study from different approaches:

PRINCIPLE BASED APPROACH

- Respect for autonomy requires that physicians allow patients to make their own decisions regarding medical care.
    - At a minimum, physicians should not interfere in a patient's pursuit of her own choices
    - At a maximum, physicians may have some obligation to refer and inform about the procedure

- Beneficence requires that physicians promote the welfare of their patients
    - Not clear what is the best way to deal with pain and distress, suffered by the patient.

CARE ETHICS

- Principle of fidelity or non-abandonment**:** maintaining relationships with one's patients is vital
- Physicians' want to remain available for and be involved in the patient's EOL care

PRINCIPLE OF DOUBLE EFFECT

- Distinction btw direct and indirect causation; killing and letting die; doing and merely allowing, and intending and merely foreseeing an outcome of one's actions
- There is strong disagreement between real-life experience of HCW (guilt) ≠ professional societies positions (equivalence) ≠ bioethics literature (debate)

ETHICS OF RESPONSIBILITY

- No clear line when a certain act (informing, prescribing, referring, etc) implies moral complicity
- The level of participation one may found acceptable/problematic depends on the category of objections
    - religious beliefs, professional obligations, deontology, consequentialism

FEMINIST AND RELATIONAL ETHICS

- Medicine is based in an ongoing relation of trust and commitment to care
- Critical points are found in several steps of the procedure, namely:
    - Disclosure of the CO; informing about the procedure; referring to a willing provider
- Ther referral compromise may be experienced as abandonment by both the physician and the patient
    - Simply referring a patient desiring AID to another willing physician may not fully adequately satisfy a physician's conscience and does not provide a neat solution as often suggested.

Real life situations challenge a simplistic understanding of CO in EOL since…

⊗ CO is not always based on an unwavering moral conviction
  → Most physicians lack a perspicuous ordering or weighting system for the values they hold

⊗ CO is not always experienced as an external conflict between physician-patient
  → It is usually an internal conflict experienced by the physician in terms of ambivalence.

⊗ Conscience does not always give rise to an objection to participating in certain medical practices.
  → A physician's conscience may instead compel her to provide certain types of care.

Therefore, CO in EOL demands an **alternative approach** that whether a physician can -or should- opt out entirely.

✓ A more nuanced understanding of the role of conscience
✓ A clearer framework for analyzing forms and degrees of moral complicity
✓ A training for clinicians designed to equip them with the skills they need:
  - Identify and evaluate their own values and moral principles
  - Navigate the diverse situations that arise in medical practice and put these values in tension
  - Develop strategies for alleviating this tension and mitigate the resulting moral distress
